# Two Sides of the Same Coin—Mechanistic Insight, Diagnostic Application and Therapeutic Translation of Bacterial and Host‐Derived Extracellular Vesicles

**DOI:** 10.1002/jex2.70093

**Published:** 2025-10-29

**Authors:** Philipp Arnold, Fanni Annamária Boros, Jochen Mattner, Gerald Seidel, Chaofan Liu, Inga Viktoria Hensel, Jan Van Deun, Raphael Schwendner, Janina Müller‐Deile, Nina Sopel, Andreas Ramming, Mario R. Angeli, Simon Rauber, Cláudia Vilhena, Andreas Baur, Stefan Wirtz, Klaus Überla, Heiko Reutter, Linda‐Marie Mulzer, Alina C. Hilger, Friederike Zunke, Wei Xiang, Gregor Fuhrmann, Claudia Günther

**Affiliations:** ^1^ Institute of Functional and Clinical Anatomy Friedrich‐Alexander‐Universität Erlangen‐Nürnberg (FAU) Erlangen Germany; ^2^ Department of Molecular Neurology University Hospital Erlangen, Friedrich‐Alexander‐Universität Erlangen‐Nürnberg (FAU) Erlangen Germany; ^3^ Mikrobiologisches Institut ‐ Klinische Mikrobiologie, Immunologie und Hygiene University Hospital Erlangen, Friedrich‐Alexander‐Universität Erlangen‐Nürnberg (FAU) Erlangen Germany; ^4^ FAU Profilzentrum Immunmedizin (FAU I‐MED) Friedrich‐Alexander‐Universität Erlangen‐Nürnberg (FAU) Erlangen Germany; ^5^ Professur für Mikrobiologie, Department Biologie Friedrich‐Alexander‐Universität Erlangen‐Nürnberg (FAU) Erlangen Germany; ^6^ Department of Medicine 1 University Hospital Erlangen, Friedrich‐Alexander‐Universität Erlangen‐Nürnberg (FAU) Erlangen‐Nürnberg Germany; ^7^ Department of Dermatology University Hospital Erlangen, Friedrich‐Alexander‐Universität Erlangen‐Nürnberg (FAU) Erlangen‐Nürnberg Germany; ^8^ Department of Medicine 4 ‐ Nephrology and Hypertension University Hospital Erlangen, Friedrich‐Alexander‐Universität Erlangen‐Nürnberg (FAU) Erlangen Germany; ^9^ Department of Internal Medicine 3 – Rheumatology and Immunology University Hospital Erlangen, Friedrich‐Alexander‐Universität Erlangen‐Nürnberg (FAU) Erlangen Germany; ^10^ Deutsches Zentrum Immuntherapie Erlangen Germany; ^11^ Department of Biology, Bacterial Interface Dynamics Junior Research Group Friedrich‐Alexander‐Universität Erlangen‐Nürnberg (FAU) Erlangen Germany; ^12^ Harald zur Hausen Institute of Virology University Hospital Erlangen, Friedrich‐Alexander‐Universität Erlangen‐Nürnberg (FAU) Erlangen Germany; ^13^ Department of Pediatrics and Adolescent Medicine University Hospital Erlangen Friedrich‐Alexander‐Universität Erlangen‐Nürnberg (FAU) Erlangen‐Nürnberg Germany; ^14^ Research Centre New Bioactive Compounds (FAU NeW) Friedrich‐Alexander‐Universität Erlangen‐Nürnberg (FAU) Erlangen Germany; ^15^ Department of Biology, Pharmaceutical Biology Friedrich‐Alexander‐Universität Erlangen‐Nürnberg (FAU) Erlangen Germany

## Abstract

Extracellular vesicles (EVs) have gained increasing attention in recent years due to their pivotal role in both health and disease. Emerging from both eukaryotic and prokaryotic cells, EVs serve as essential mediators of intercellular communication, exceeding the simplistic interactions observed with individual molecules. In this comprehensive review, we will focus on both Bacterial Extracellular Vesicles (BEV) and on Host derived Extracellular Vesicles (HEV) and highlight mechanistic principles, as well as their transformation into diagnostic and therapeutic tools. We will start with a short introduction into the biogenesis and principal properties of BEV and HEV. We will then focus on the composition of BEV and introduce OMICs‐based studies that helped to unravel their complex constitution. As both BEV and HEV interact with different epithelial and endothelial barriers and shape their properties, we will highlight mechanistic principles for both EV types. Starting from the intestinal system, where we will look at BEV and how these BEV overcome the intestinal barrier to change distant organs and the patient's immune system. We will then visit other endothelial and epithelial sites of the human body and summarize how HEV shapes these barriers and how HEV can overcome these barriers. We will then turn towards diagnostic and therapeutic approaches. As both BEV and HEV are currently suggested as diagnostic markers and are being investigated as potential therapeutic agents. Lastly, we will discuss current challenges and provide an outlook on the future in the field. This review seeks to raise awareness for both bacterial and host‐derived EVs, highlighting that they present two sides of the same coin.

## 1 Preface

Extracellular vesicles (EV) emerge as pivotal mediators of intercellular, inter‐organ and inter‐kingdom communication. EVs are membrane‐enclosed structures, ranging in size from 30 to ∼350 nm, and originate from virtually all cells of all kingdoms of life including microbial organisms (Xie, Haesebrouck, et al. 2023; Toyofuku et al. 2019, 2023; Díaz‐Garrido et al. 2021; Tiku and Tan 2021; Kaparakis‐Liaskos and Ferrero 2015). Virtually all cells of the human body, including immune cells, neurones, and epithelial/endothelial cells, release EVs and thus EVs add an additional dimension to the already complex landscape of host‐microbe interaction and communication. Their significance extends beyond traditional cellular boundaries, as they orchestrate a wide array of biological processes, such as immune regulation, tissue repair and neuronal signalling. Besides their physiological role, they have been identified as disease‐associated in various pathologies, including cancer, neurodegenerative disorders and immune‐mediated inflammatory diseases.

The versatile interactions of host‐ and microbiota‐derived EV attract more fascination to this field of research. Microbiota‐derived EVs originate from the vast and intricate world of commensal microorganisms residing on inner (muco‐epithelial) and outer (skin) surfaces of the host. These vesicles, released by bacteria, fungi and other members of the microbiota, extended our understanding of the dynamic interplay between the host and its symbiotic microbial communities. Microbiota‐derived EVs carry cargo that mirrors their microbial origin, offering a unique channel for microbe‐host communication, which influences host physiology and pathology.

**FIGURE 1:**
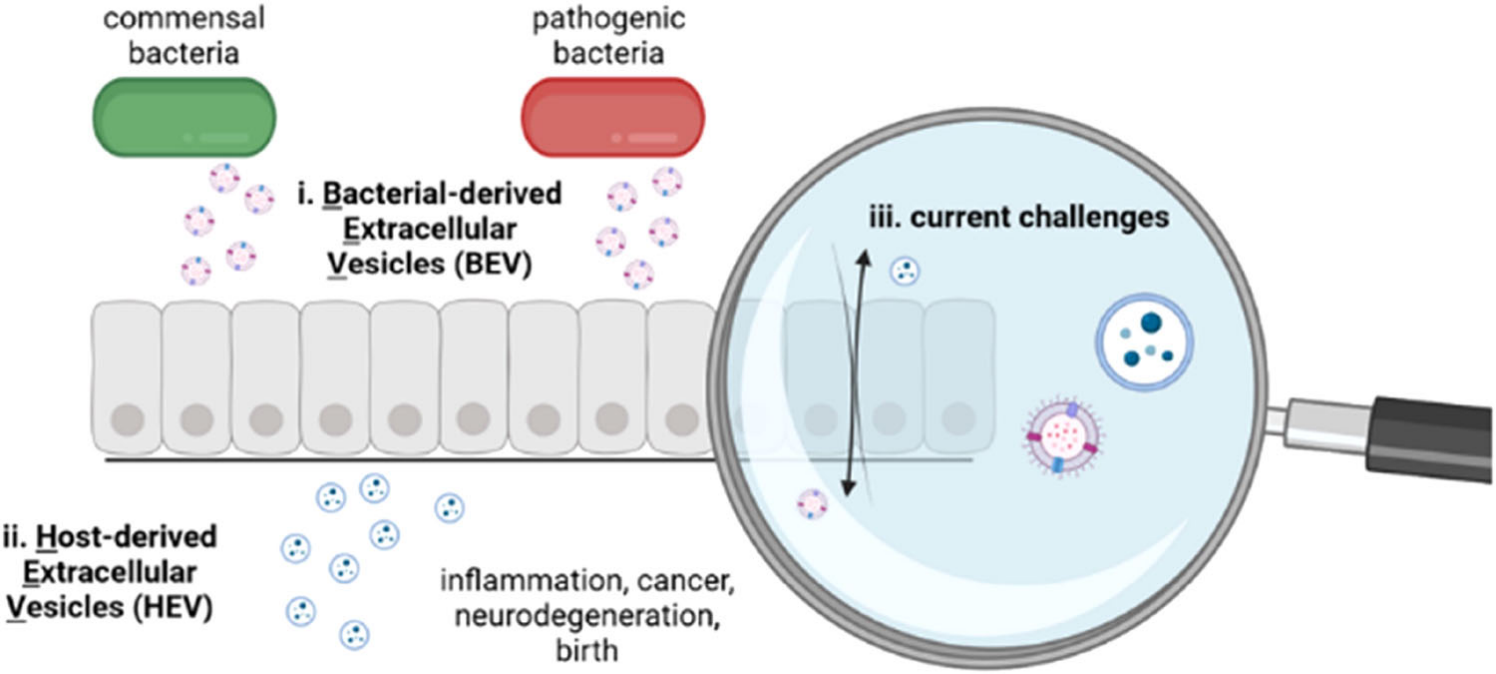
Interaction of different EV populations with barriers. Commensal and pathogenic bacterial extracellular vesicles (BEV), as well as host‐derived extracellular vesicles (HEV), interact with endothelial and epithelial barriers of the host system.

One feature shared by microbial and host derived EVs, is their ability to interact with and cross barriers of the human body (e.g., epithelial and endothelial barriers, Figure 1), which are typically impermeable for cells. Thus, EVs represent a powerful tool for all cells to communicate across borders. In this review, we will highlight some important aspects of EV biology with a focus on mechanistic aspects of how EV populations interact with human barriers and elaborate on their potential as diagnostic tools and therapeutic agents.

## 2 Introduction to BEV and HEV: Biogenesis and Differentiation

### 2.1 Bacterial Extracellular Vesicles (BEV)

**FIGURE 2:**
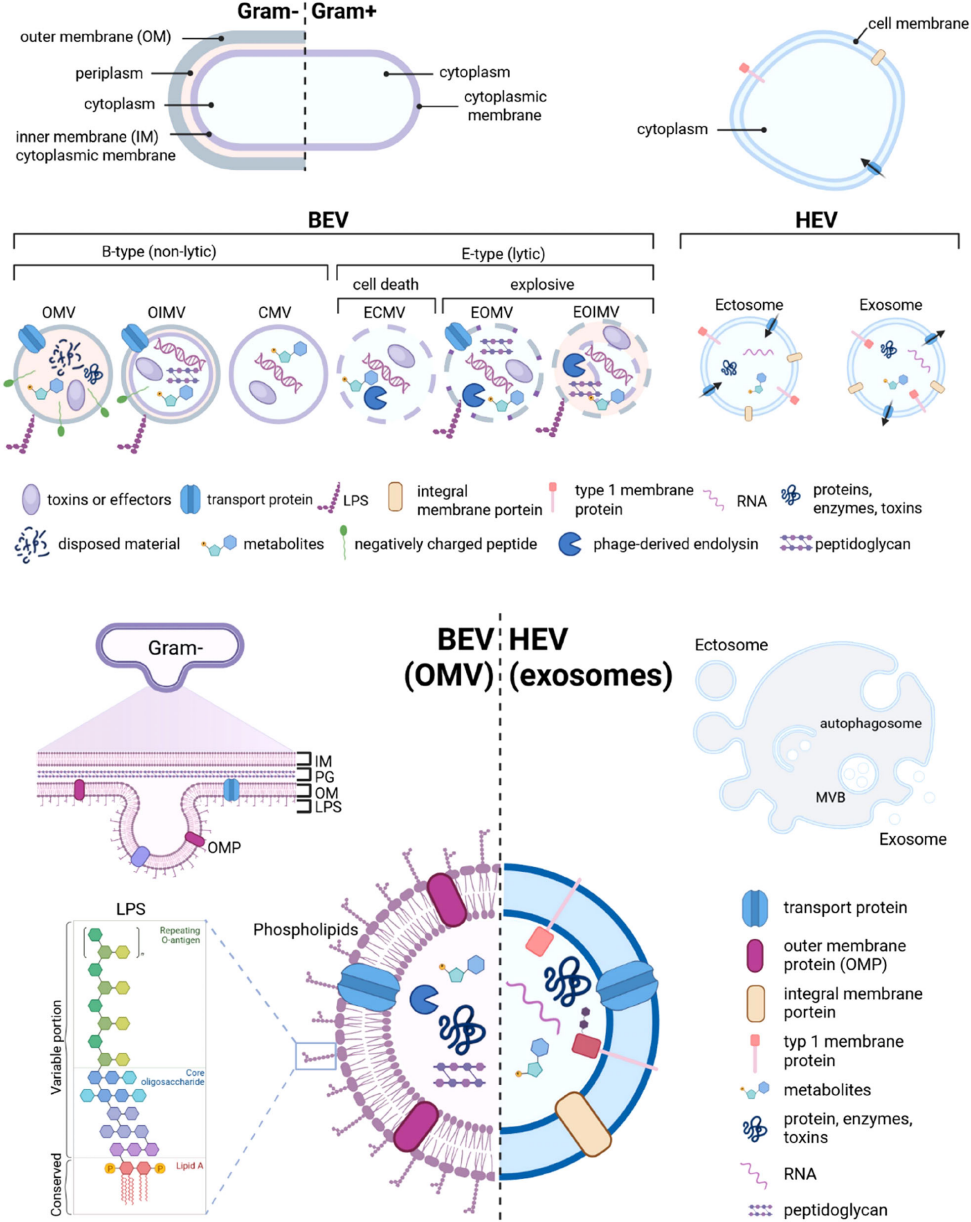
Schematic overview of EV biogenesis. This graphic illustrates the diversity of EV derived from bacteria and host cells. Bacterial‐derived EVs (BEVs) originate via blebbing of the membrane (b‐type) and include outer membrane vesicles (OMV, gram‐negative), outer‐inner membrane vesicles (OIMV, gram‐negative), and cytoplasmic membrane vesicles (CMV, gram‐positive bacteria). BEV produced via explosive cell death (E‐type) include explosive cytoplasmic membrane vesicles (ECMV, gram‐positive bacteria), explosive outer membrane vesicles (EOMV, gram‐negative bacteria) and explosive outer‐inner membrane vesicles (EOMV, gram‐negative bacteria). Host‐derived exosomes originate from the endosomal pathway (multi‐vesicular bodies; MVB), and ectosomes, also known as shedding microvesicles, are directly released from the plasma membrane through outward budding. Both, BEV and HEV, contain different types of molecules that represent their mother cell and include proteins, metabolites, sugars, lipids and nucleic acids.

Within the network of bacterial communication, BEV play a pivotal role. In general, all bacteria, gram‐positive and gram‐negative produce EV and release these into their environment. The outer membrane‐derived lipid bilayer is a fundamental structural component of BEV, providing stability and encapsulating the vesicular lumen. Depending on the formation of BEV, they differ in their membrane composition and cargos. Both, commensal and pathogenic bacteria actively produce these vesicles as part of their regular growth processes and in response to various stress factors. Based on their biogenesis, they can be categorized into different types (Toyofuku et al. 2019, 2023; Juodeikis and Carding 2022). In contrast to gram‐negative bacteria, which will be discussed below in more detail, the biogenesis of EVs from gram‐positive bacteria is fundamentally different due to the absence of an outer membrane and the presence of a thick peptidoglycan layer. Despite these structural differences, gram‐positive bacteria actively release EVs, often referred to simply as cytoplasmic membrane‐derived vesicles. Their formation is thought to involve localized cell wall remodelling, surfactant‐mediated weakening of the peptidoglycan, or turgor‐driven extrusion. A key difference between gram‐positive and gram‐negative BEVs lies in their membrane origin and cargo composition. While gram‐negative BEVs derive from the outer membrane and are enriched in lipopolysaccharide (LPS), outer membrane proteins, and periplasmic contents, gram‐positive BEVs originate from the cytoplasmic membrane and lack LPS but may carry lipoteichoic acids and a distinct set of immunomodulatory proteins and metabolites. As we will put a focus on gram‐negative‐derived BEV and, in particular, outer membrane vesicles (OMV) in the following chapters, we refer you to a different review for more detailed information on gram‐positive bacteria‐derived EV (e.g. Brown et al. 2015). Gram‐negative bacteria produce three different types of EV: (i) OMV, generated by nonlytic blebbing of the outer membrane; (ii) Explosive Outer Membrane Vesicles (EOMV), generated by lytic release of the outer membrane; and (iii) Outer Inner Membrane Vesicles (OIMV), generated by co‐release of the inner and outer membranes (Figure 2). Among non‐lytic BEV generated by gram‐negative bacteria, OMV stand out as the predominant type (Kaparakis‐Liaskos and Ferrero 2015; Schwechheimer and Kuehn 2015). OMVs are small, spherical structures, typically ranging in size from approximately 20 to 250 nm, and are primarily formed through outer membrane blebbing, a process involving the outward protrusion and subsequent pinching off of portions of the outer membrane (Toyofuku et al. 2019; Juodeikis and Carding 2022; McMillan and Kuehn 2021). LPS, phospholipids, metabolites and various outer membrane and periplasmic proteins are signature components of OMVs (Table 1). Consequently, the presentation of LPS on the outer surface of gram‐negative bacteria‐derived OMV is an important inducer of host immune response (Tiku and Tan 2021). Specific mechanisms governing the release and variability of BEV remain mainly poorly understood. For the gram‐negative gut commensal *Bacteroides thetaiotaomicron (B. theta)*, first data suggest specific release of distinct BEV populations during different growth phases (Juodeikis et al. 2024). Early‐stage vesicles were found to be predominantly composed of OMV, featuring a higher concentration of lipoproteins. In contrast, vesicles released during the late phase were predominantly composed of lytic vesicles, unveiling an enrichment of cytoplasmic proteins. Undoubtedly, additional research efforts are needed to unveil principles for BEV release and to identify stimuli that trigger the release of specific BEV subpopulations.

**TABLE 1:** Comparative features of bacterial extracellular vesicles (BEV) and host‐derived extracellular vesicles (HEV).

*Feature*	*BEV (OMV)*	*HEV*
***Origin***	*Outer membrane*	*Cell membrane (ectosomes)* *Multivesicular bodies (exosomes)*
***Size***	*80–200 nm*	*50–350 nm*
***Cargo***	*Proteins (e.g., OMPs), lipids (e.g., LPS), metabolites, virulence factors (genetic material)*	*Proteins, lipids, mRNA, microRNA, amino acids, sugars*
***Lipid composition***	*Phospholipids, sphingolipids, glycerophospholipids, phosphatidylglycerol, phosphatidylethanolamine, lipoproteins, lipopolysaccharides, lipooligosaccharides, cardiolipin*	*Represents the mother cell, might have asymmetry*
***Surface marker***	*LPS, OmpC; not fully identified*	*CD9, CD81, ADAM10, CD63; may also be influenced by cell of origin*
***Matrix***	*Faeces, serum, CSF, synovial fluid, bronchoalveolar lavage fluid*	*All body fluids, faeces*

*Note*: Table summarizing the distinguishing features between BEV represented by outer membrane vesicles (OMV) and HEV.

### 2.2 Host Extracellular Vesicles (HEV)

Virtually all cells of the human body release EV under physiological and pathological conditions. These HEVs come in two fundamentally different flavours with regard to their biogenesis and present themselves as ectosomes (100–350 nm) or exosomes (50–100 nm) (Cocucci and Meldolesi 2015). Additionally, EVs can be discriminated into small EVs (< 200 nm) and large EVs (> 200 nm). This is especially encouraged if the biogenesis of EV is unclear. All EV populations are released by living cells and must be differentiated from apoptotic bodies, which are membrane‐enclosed cellular remains (often > 500 nm) of cells after regulated cell death. In the following, we will highlight some differences between exosomes and ectosomes, as these derive from fundamentally different events. Ectosomes derive from budding events of the cell membrane and display transmembrane proteins in their natural orientation towards extracellular space. Exosomes, on the other hand, originate from endocytic vesicles that are internalized into autophagosomes, which subsequently form multi‐vesicular bodies (MVB). These MVB can then fuse with the cell membrane and release the vesicles as exosomes into extracellular space. Although the general view is that protein orientation of HEV‐associated proteins is as on the cell membrane, there is data that this is not exclusively the case (Figure 2) (Cvjetkovic et al. 2016). Both HEV populations, ectosomes and exosomes, are often equated as one, due to the limited availability of methods to separate both. However, the difference in protein orientation within the membrane needs to be accounted for when immunoprecipitating HEV from biofluids. Additionally, shedding substrates of proteases, including the IL‐6 receptor, CD109 and the prion protein (Lückstädt et al. 2021; Arnold et al. 2020; Linsenmeier et al. 2018), can only be released into extracellular space from those EVs with a parental cell‐like protein orientation.

## 3 OMV at the Mucosal Intestinal Barrier and Beyond

The mammalian gastrointestinal tract (GI) represents a complex and dynamic ecosystem that plays a pivotal role in the digestion of food, nutrient absorption and the regulation of various physiological processes within the human body. Beyond these primary functions, the gastrointestinal tract also serves as a frontline interface where the human host interacts with numerous environmental factors. Among these, one of the most intriguing and influential interactions is the symbiotic relationship between the gut cells and resident microbiota, comprising trillions of microorganisms collectively known as the gut microbiota. This symbiosis between the host and its microbial inhabitants has a profound impact on both health and disease, shaping the overall well‐being of an individual locally in the gut, but also systemically.

### 3.1 OMV in the Gut and Their Connection to Other Tissues

OMV are present throughout the GI tract. Bacterial species, local microbiota and environmental conditions influence their distribution along its length (Bittel et al. 2021; Jones et al. 2020). Thus, OMV are not only vehicles for communication, but also tools for microbial competition. Bacterial species may strategically release OMV in specific regions of the GI‐tract to outcompete others for (nutritional) resources or to establish dominance in certain niches. This might be particularly relevant during bacterial infection. Furthermore, environmental factors, such as diet, as well as host genetics, can influence the biodistribution of OMV within the GI‐tract. As an example, dietary changes can alter the microbial composition in different parts of the GI tract and thereby influence the types and abundance of OMV released. Similarly, host genetics may influence the distribution of specific bacterial species, which in turn affects the OMV profile across the GI tract.

While the localization of OMV within specific GI regions is a dynamic and context‐dependent process, several reports also indicate their presence in different extra‐intestinal tissues (Bittel et al. 2021; Jones et al. 2020; Tulkens, Vergauwen, et al. 2020; Xie, Cools, et al. 2023). This implies that OMV must be able to cross the mucosal barrier under certain conditions. Indeed, we and others have shown that OMV travel significant distances from their parent bacteria located in the gut lumen (Jones et al. 2020). Moreover, there is emerging evidence that OMV can be detected in human biofluids and tissues, and a recent study reported increased serum levels of LPS‐positive EV in patients with inflammatory bowel disease (Tulkens, Vergauwen, et al. 2020). These findings strongly suggest that OMV cross the mucosal and also the blood barrier. Understanding how OMV are distributed within the GI tract and their ability to cross mucosal barriers is of significant interest due to their potential implication for host‐microbe interactions, health and disease as outlined below.

**FIGURE 3:**
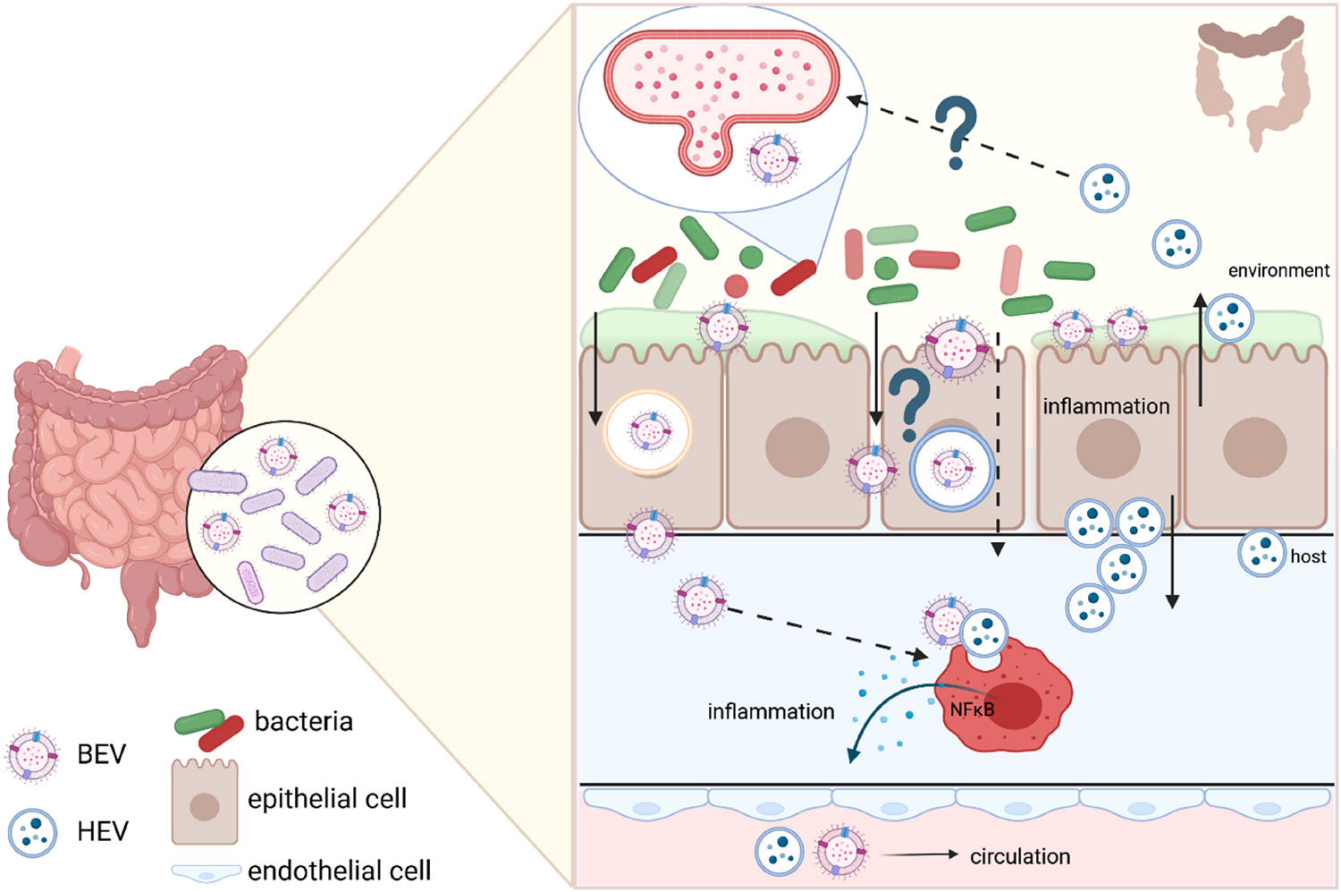
Host‐microbial crossroads within mucosal tissues: Entry mechanisms for BEV on the example of the gut. Originating from the gut microbiota residing in the luminal environment of the gastrointestinal tract, BEV play a crucial role in the intricate cross‐talk between commensal/pathogenic microbes and the host. BEV can engage various routes to traverse the intestinal barrier, including endocytosis or, particularly noteworthy during inflammatory states, translocation through the intercellular space. Upon endocytosis, intestinal epithelial cells can release bacterial antigens encapsulated within exosomes, representing a unique subset of host EV enriched with bacterial cargo. This mechanism underscores the bidirectional exchange of molecular information between microbial and host cells within the gut milieu. In this system, HEV contribute to this intricate interplay by exerting regulatory effects on the gut microbiota. HEV can be released into the gut lumen, where they modulate microbial composition and function, thus influencing host‐microbiota homeostasis.

To cross the mucosal barrier, OMV must initially penetrate the protective mucus layer that coats the intestinal epithelium (Figure 3). This mucus layer primarily consists of mucins, water, electrolytes, and antimicrobial peptides, forming a dynamic barrier that regulates microbial interactions and protects the underlying epithelial cells. Interestingly, both in vivo and in vitro experiments utilizing intestinal organoids have revealed that OMVs are taken up by intestinal stem cells located in the crypt region—a relatively sterile compartment of the GI (Bittel et al. 2021). This finding suggests that the mucus layer, a gel‐like substance covering the intestinal epithelium, might function as a transport medium, facilitating the translocation of OMV from the gut lumen to the bacteria‐free crypt base. Importantly, the mucus layer does not only trap potential threats but also harbours a rich ecosystem of beneficial microorganisms that contribute to overall gut health. Interestingly, *B. theta*, one of the most abundant bacterial commensals found in the human colon, produces a variety of enzymes that degrade carbohydrates within the mucus layer, and OMVs produced by this species contain some of these enzymes (Elhenawy et al. 2014; Martens et al. 2008). One of these enzymes packed in OMVs is sulphatase, an enzyme that is capable of cleaving sulphate groups from complex mucin glycoproteins found in the mucus layer. This allows *B. theta* to break down and utilize mucins as a nutrient source and to overcome the mucosal layer to directly interact with the intestinal epithelial cells (Hickey et al. 2015). The intestinal epithelium is a single layer of epithelial cells that helps us to efficiently digest and take up nutrients, but at the same time separates the gut lumen harbouring the gut environment from the host. Accordingly, its tight junctions ensure that only specific substances are allowed to pass, a process essential for nutrient absorption and immune surveillance. Previous studies have demonstrated that OMV translocation across this layer involves both paracellular and transcellular pathways and is strongly enhanced under inflammatory conditions (Krsek et al. 2023). For transcytosis across epithelial cells, OMV exploit the host's endocytic machinery. This process involves vesicle uptake from the luminal side, transport through the cell, and release on the basolateral side, allowing OMV to access the underlying mucosal tissue. In non‐phagocytic cells, various primary mechanisms exist for the uptake of small solutes, including actin‐dependent macropinocytosis, clathrin‐mediated endocytosis, caveolin‐mediated endocytosis or clathrin‐ and caveolin‐independent mechanisms such as membrane fusion or lipid raft formation (Bittel et al. 2021; Jones et al. 2020; Jan 2017; Rewatkar et al. 2015) and reviewed in (O'Donoghue and Krachler 2016). Dynasore, a dynamin inhibitor, can block clathrin‐ and caveolin‐mediated endocytosis. Indeed, studies have demonstrated that dynamin inhibition is associated with a reduced uptake of OMV into intestinal epithelial cells, suggesting that this uptake mechanism contributes to the translocation of OMV across the gut barrier. Importantly, two recent studies emphasized that the internalization of OMV by epithelial cells is crucial for their immunomodulatory activities (Bittel et al. 2021; Jones et al. 2020). Accordingly, inhibition of OMV uptake resulted in the suppression of cytokine induction (Thapa et al. 2023). OMV can also transmigrate through epithelial cells via a paracellular route by modulating the integrity of tight junctions between epithelial cells (Jones et al. 2020). Notably, OMV released by certain bacteria can also contain components that have the ability to disrupt tight junctions, potentially facilitating the passage of both OMV and other substances through the intestinal epithelium. The components in OMV responsible for disrupting tight junctions can vary depending on the bacterial species and strain (Krsek et al. 2023). *Campylobacter jejuni* OMV cleave critical adherent's junction proteins like E‐cadherin and the tight junction protein occludin (Elmi et al. 2016). In the context of *Helicobacter pylori*, OMV containing the cytotoxin CagA localize in close proximity to cellular junctions, resulting in the redistribution of the tight junction protein ZO‐1 (zonula occludens‐1) into the cytoplasm (Turkina et al. 2015). Although research on commensal OMV‐host cell interactions remains relatively scarce, an intriguing finding involves the probiotic strain *Escherichia coli* (*E. coli*) Nissle 1917, which has been observed to up‐regulate the expression of barrier‐reinforcing proteins such as ZO‐1 and ZO‐2 (Alvarez et al. 2016). Collectively, these investigations indicate that OMV can exert direct or indirect effects on the composition of junctional complexes, facilitating their ability to traverse epithelial barriers, a mechanism often associated with microbial virulence.

### 3.2 Role in Host Immune System

Once OMV have crossed the intestinal epithelium, they can engage with mucosal immune cells. This interaction is a highly dynamic and complex process that plays a crucial role in the host‐microbial relationship, immune surveillance and overall gut health. OMV play a dual role in this interaction: on the one hand, they can help to promote immune tolerance, preventing the immune system from overreacting to harmless antigens present in the gut. On the other hand, they can stimulate the immune system to respond appropriately to potential pathogens. Since OMV carry various bacterial antigens, it is not surprising that they can activate immune responses by triggering pattern recognition receptors such as Toll‐like receptors (Tiku and Tan 2021; Kaparakis‐Liaskos and Ferrero 2015). This activation can lead to the production of pro‐inflammatory cytokines and chemokines, which recruit immune cells to the site of infection or interaction (Bittel et al. 2021; Ismail et al. 2003). Antigen‐presenting cells such as dendritic cells can also present OMV cargo to T cells. This antigen presentation can influence adaptive immune responses, including the activation of specific immune memory cells (Cheng et al. 2021; Durant et al. 2020). In this context, OMV from commensal bacteria have been shown to promote immune tolerance. They may induce regulatory T cells and suppress inflammatory responses, and thus, help to maintain mucosal immune homeostasis. Indeed, this tolerance induction prevents excessive inflammation and autoimmune reactions. This is also relevant during the early stages of postnatal life, in which the gut microbiota undergo dynamic changes in both their structural and functional composition. This postnatal period represents a critical window where OMV may help to educate and modulate the host's immune system, contributing to the development of an effective defence against pathogens and the promotion of immune homeostasis (Turunen et al. 2023; Shen et al. 2012). For example, *Bacteroides fragilis* releases a polysaccharide (PSA) in OMV that induces immunomodulatory effects and prevents experimental mucosal inflammation. Dendritic cells sense OMV‐associated PSA through TLR2, resulting in enhanced regulatory T cell frequency and anti‐inflammatory cytokine production (Shen et al. 2012). Thereby the gut commensal *Bacteroides fragilis* produces OMV with immunomodulatory functions that protect against colitis. Mechanistically, OMV require inflammatory bowel disease‐associated genes, ATG16L1 and NOD2, to activate a noncanonical autophagy pathway during protection from colitis (Chu et al. 2016). ATG16L1‐deficient dendritic cells do not induce regulatory T cells to suppress mucosal inflammation. Importantly, immune cells from human subjects with a major risk variant in ATG16L1 are defective in T(reg) responses to OMV. These experimental data, combined with the clinical background, may implicate those polymorphisms in susceptibility genes that promote disease through defects in ‘sensing’ protective OMV from the microbiome. Besides *Bacteroides fragilis*, several other studies have shown that OMV can mimic the anti‐inflammatory properties of their parent bacteria. For instance, probiotic bacteria like *E. coli* Nissle 1917 (EcN) have demonstrated efficacy in inducing and maintaining remission of inflammatory conditions such as ulcerative colitis. Notably, purified EcN OMV can replicate these beneficial effects when administered orally (Fábrega et al. 2017). They can ameliorate mucosal injury, reduce inflammation, and normalize cytokine expression, mirroring the therapeutic impact of the live probiotic. This suggests that OMV might provide a safer alternative to traditional probiotic administration.

While this highlights the powerful nature of OMV in educating the host immune system, human pathobionts have evolved strategies to adapt to host defence and to evade immune detection. For example, the human pathogen *Vibrio cholerae* increases OMV release in the early phase of infection (Zingl et al. 2020). Hypervesiculation allows faster adaptation to host defence mediators and enhances intestinal colonization fitness and inflammation. OMV produced by the bacterium *B. theta* also contribute to intestinal inflammation, particularly in genetically susceptible mice. *B. theta* antigens access host immune cells via OMV containing bacterial enzymes able to degrade the mucus layer. Once OMV have crossed the mucosal barrier, they interact with local macrophages to mediate mucosal inflammation (Hickey et al. 2015). This research underscores the potential importance of understanding the interactions between commensal bacteria like *B. theta* and the host's immune system in the context of health and disease. While these data demonstrate the impact of OMVs on mucosal inflammation, another recent study provides insights into the potential involvement of OMVs derived from *Fusobacterium nucleatum* in the pathogenesis of rheumatoid arthritis (RA), suggesting a role of these shuttle systems along the gut‐joint axis (Hong et al. 2023). They discovered that OMV of *Fusobacterium nucleatum* containing the virulence determinant FadA travel into the joints, triggering a local inflammatory response by interacting with synovial macrophages. Interestingly, OMV containing FadA were also elevated in the synovium of RA patients as compared to controls. These findings suggest that *Fusobacterium nucleatum*‐derived OMV may play a causal role in worsening RA symptoms and provide potential therapeutic targets for mitigating RA clinically. Another study demonstrating that *E. coli*‐derived OMVs induce immune cell activation and progression of cirrhosis in the liver of mice highlights the proinflammatory nature of OMVs along the gut‐liver axis (Natsui et al. 2023). This study further provides the first evidence that OMV are present in the ascites of patients with cirrhosis, as indicated by the fact that EV isolated from this biofluid were positive for bacterial membrane markers, such as outer membrane proteins. Another recent study investigated the influence of gastrointestinal bacteria, particularly *Helicobacter pylori*, a common gastric pathogen on the pathogenesis of Alzheimer's disease to visualize a role in gut‐brain communication (Xie, Cools, et al. 2023). Interestingly, *Helicobacter pylori* infection has been associated with an increased risk of developing Alzheimer's disease (Albaret et al. 2020). Preclinical studies have now demonstrated that *Helicobacter pylori* OMV can breach biological barriers, reaching the brain, where they are internalized by astrocytes. This leads to glial cell activation, neuronal dysfunction, amyloid‐β pathology, and cognitive decline, key features of Alzheimer's disease. Similarly, a second study demonstrated that oral or intravenous OMV administration allowed OMV to reach the brain, alter astrocyte function and promote neuronal damage in vivo (Palacios et al. 2023). Thus, OMV do not only target cells within the gut, but also serve as efficient shuttle systems, delivering bioactive molecules and signals to distant organ systems that lack direct exposure to environmental factors. These recent studies further demonstrate that OMV can also replicate pro‐inflammatory effects associated with certain bacteria, which induce strong pro‐inflammatory responses in host immune cells. This can be advantageous in situations where immune activation is required. Understanding how specific OMV trigger these responses may open doors to therapeutic applications, such as vaccine development or targeted immune modulation, as discussed below.

## 4 Host‐Derived Extracellular Vesicles (HEV)

### 4.1 HEV and Their Role at the Endothelial Barrier

**FIGURE 4:**
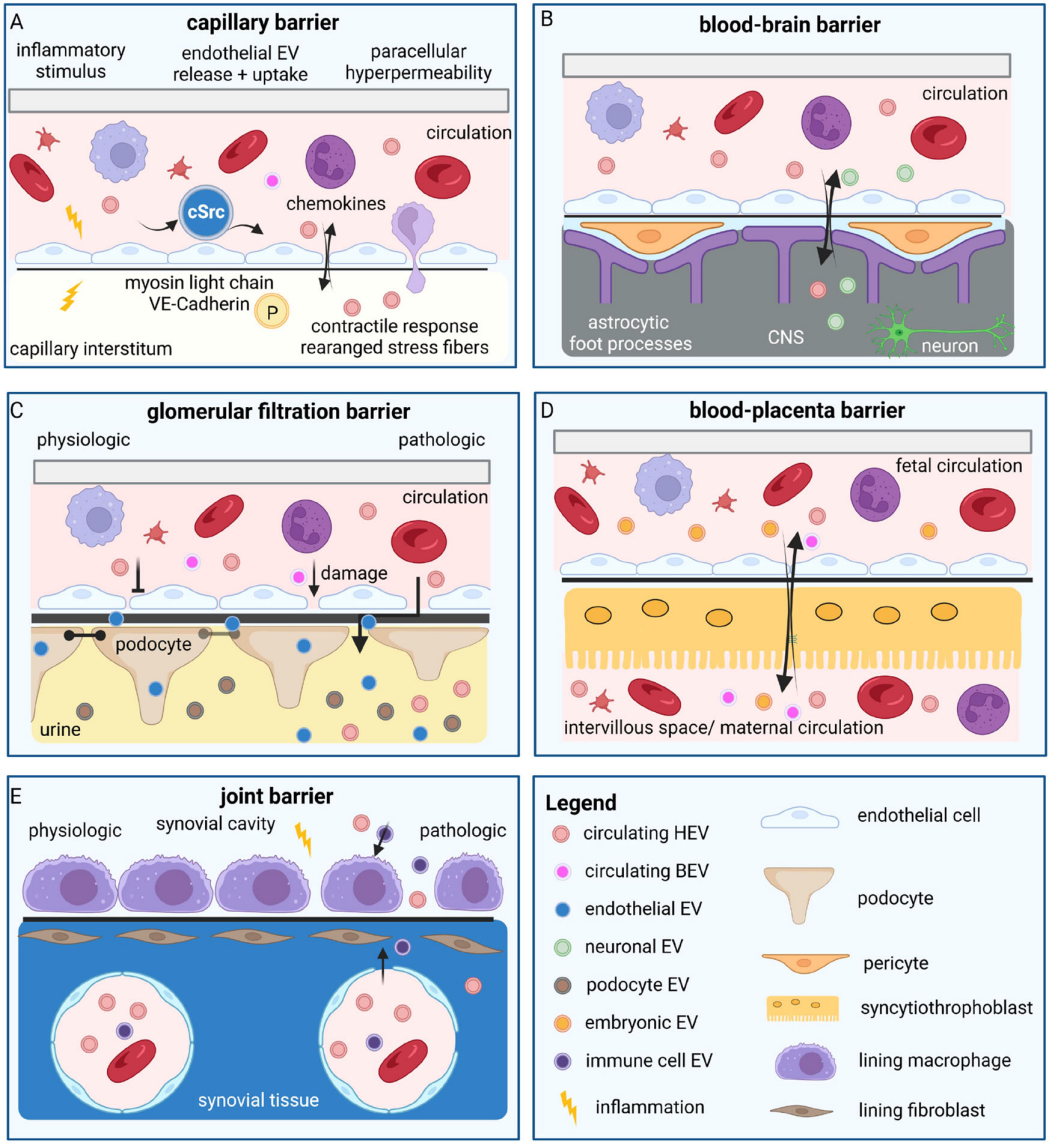
EV at different endothelial and epithelial barriers. (A) The capillary barrier consists of a single layer of endothelial cells sitting on a basement membrane. Endothelial‐derived EV (blue) contain the protein kinase cSrc and change the barrier function of other endothelial cells. This makes the barrier more penetrable for immune cells, and circulating HEV (red) can cross the endothelium and reach the periphery. Conversely, peripheral chemokines can also cross the endothelial barrier more easily, attracting additional circulating immune cells. (B) The blood‐brain barrier (BBB) is a complex multicellular structure, consisting of tightly connected endothelial cells, pericytes, a basement membrane, and astrocyte end‐feet. The term ‘barrier’ is not precise, however, since the BBB functions as a semipermeable membrane, strictly regulating the interplay and communication between the peripheral circulation and central nervous system (CNS). Besides the bi‐directional passage of various micro‐ and macro‐molecules, EVs have also been described to enter the CNS from the bloodstream and vice versa, thus ensuring a further mode of connection between the periphery and the CNS both in health and disease. (C) The glomerular filtration barrier (GFB) is composed of glomerular endothelial cells lining the renal arterioles, the glomerular basement membrane and podocytes facing the urinary space. Under physiological conditions, circulating HEV and BEV are predominantly retained in the bloodstream. However, when the GFB is leaky, due to pathological changes, EV can cross the GFB and can act on downstream cells, such as podocytes and cells of the tubular system, or they will be lost with the urine and could serve as biomarkers for renal damage. (D) The blood‐placenta barrier is formed by the syncytiotrophoblasts I and II (depending on the time past implantation) and the tight foetal endothelium. In later stages of the embryonic development, only one layer of the syncytiotrophoblast remains, and the diffusion distance reduces to 3.5 µm. EV from maternal and foetal origin might cross the blood‐placenta barrier. (E) The joint barrier comprises two compartments, the synovial lining layer and a synovial sublining layer consisting of fibroblasts, macrophages and endothelial cells, both crucial for joint health. Dysregulation of synovial barrier integrity in inflammatory or degenerative conditions involves HEV derived from resident and infiltrating cells modulating inflammation and tissue remodelling processes.

The endothelial blood barrier presents the largest barrier within the human body. It covers an area of approximately 600 m^2^ and changes its properties depending on the local function. The endothelial cells can form a tight (e.g., in the brain), discontinuous or fenestrated endothelial barrier (e.g., liver, intestine). Both discontinuous and fenestrated endothelia have openings with a diameter between 100 and 200 nm, thus allowing different EV populations to pass through them freely. With a size of approximately 50–70 nm, small EVs should pass through these physiological endothelial openings without hinderence. However, there might be molecular mechanisms that foster EV transport across the endothelial barrier as HEV modulate inflammatory conditions such as arteriosclerosis or local inflammatory processes (Adamczyk et al. 2023; Charla et al. 2020) (Figure 4A). Specific mechanisms by which they reach the periphery and extravasate from the capillary lumen remain elusive. Similar to cargo, HEV could take the paracellular or transcellular route. Adding to the complexity, endothelial cells themselves release EV upon stimulation with pro‐inflammatory stimuli such as TNFα, IL‐1b, LPS or C‐reactive protein (Hromada et al. 2017). This induces (i) the activation of leukocytes (to enter the site of inflammation) and (ii) a rearrangement of cell‐cell contacts between endothelial cells, which leads to paracellular hyperpermeability. cSrc seems to orchestrates the rearrangement of cell‐cell contacts, as HEV depleted of cSrc failed to induce such rearrangement. On the molecular level, myosin light chain and VE‐cadherin undergo phosphorylation, which leads to a contractile response and the formation of actin stress fibres (Chatterjee, Yang, Ma, et al. 2020) (Figure 4A). Whether this is the only molecular mechanism induced by cSrc transferred through HEV is not clear, as cSrc signals upstream of multiple intracellular signalling cascades, including the MAP kinase (e.g., pro‐inflammatory) and β‐catenin pathways, and induces endothelial to mesenchymal transition (Kim et al. 2019). Thus, endothelial‐derived HEV seem to activate an autocrine effect that induces a (local) leaky endothelial barrier, which might allow a better diffusion of HEV, cytokines and chemokines that attract immune cells. Another question with regard to HEV generated by endothelial cells is their site of release. Whether there are differences between apically and basolaterally released HEV is unclear, similar to whether there are functional aspects of such vesicles. Besides endothelial cell‐derived HEV, tumour‐derived EV (TEV) were shown to change endothelial barrier function (Guo et al. 2023). HEVs stemming from tumour cells carry different active substances (e.g. miRNAs, cell surface receptors, and cytokines) that modulate the endothelial cell barrier and thereby favour tumour cell dissemination to specific organs (Becker et al. 2016). With regard to the endothelial barrier, two different functionalities might be of importance. First, HEV can induce an inflammation‐like phenotype in the vicinity of the endothelium, which in turn could lead to the induction of the above‐described autocrine loop of endothelial vesicle release and reduced cell‐cell contact strength. This pro‐inflammatory condition could arise from the recruitment and reprogramming of bone marrow‐derived macrophages through the transfer of cMet (Peinado et al. 2012), the activation of local macrophages through the transfer of Annexin2 (Maji et al. 2017) or the activation of adjacent epithelial cells (e.g., in the lung) where HEV‐encapsulated snRNAs induce the expression of TLR3, which leads to neutrophil recruitment (Liu et al. 2016). Second, HEV can directly act on endothelial cells and thereby increase barrier permeability. This was shown for miRNA103 transferred from hepatocellular carcinoma cells via TEV, which inhibited the expression of VE‐Cadherin, p120‐catenin and zonula occludens 1 (Fang et al. 2018), diminishing the integrity of endothelial junctions. Thus, EVs from different sources can pass and modulate the endothelial barrier of blood vessels.

### 4.2 EV and the Blood‐Brain Barrier (BBB)

The interface between the brain and blood is a multicellular complex structure, the BBB, created by endothelial cells connected tightly *via* inter‐endothelial junctional complexes, pericytes, a basement membrane, and the endfeet of astrocytes (Abdelsalam et al. 2023) (Figure 4B). As the name implies, the BBB constitutes a border; however, the term ‘barrier’ is somewhat misleading, since it rather constitutes a selective semi‐permeable membrane which strictly regulates the two‐directional passage of various micro‐ and macromolecules (Galea 2021). While the primary function of the BBB is to protect the brain from potentially damaging substances present in the blood, it also allows the entry of essential nutrients and energy substrates into the brain in order to fulfil its high energy demand. The BBB also enables the passage of signalling molecules from the blood, permitting and provoking central nervous system (CNS) responses that impact peripheral organs. The transport of molecules through the BBB from the blood to the brain and vice versa opens possibilities for identifying CNS‐derived molecules/signatures in the blood and systemic administration of compounds from the blood to the brain. Consequently, this could facilitate the recognition of biomarkers associated with brain‐related diseases and enable the delivery of drugs for the treatment of CNS disorders. However, the highly selective nature of the BBB makes it challenging to achieve accumulation of desired therapeutic substrates in the CNS. Presumably, almost all large molecular drugs and 98% of small molecule therapeutics cannot cross the BBB (Pandit et al. 2020). The tight connections within the BBB mainly allow the passage of lipid‐soluble drugs; therefore, a feasible way to increase the permeability of compounds is to improve their lipophilicity. Considering that EVs are composed of a lipid bilayer, encapsulating hydrophilic cargo molecules within these vesicles can be a promising approach for drug delivery to the CNS.

Indeed, an increasing body of evidence suggests that EVs are capable of crossing the BBB in both directions. The exact mechanisms of EV transport through the BBB are not fully understood. The most accepted view is via transcytosis, that is, crossing the intracellular cell compartment. Findings suggest that EVs are either completely transported through the endothelial cells, thus exerting their effect on the brain, or are sequestered inside the cells, leading to the modulation and regulation of transport mechanisms of the BBB (Abdelsalam et al. 2023). Using an in vitro trans‐well BBB model, Morad and colleagues found decreased EV internalization and crossing through endothelial cells upon treatment with the endocytosis inhibitor Dynasore, supporting the notion that EVs cross the BBB by an active endocytosis mechanism (Morad et al. 2019). The presence of different surface proteins on EVs, which reflect the origin of the vesicles and pathophysiological changes such as inflammation, are also strong determinants of the ability of EVs to cross the BBB. Banks et al. (2020) investigated the effect of the cellular origin on the efficiency of exosome transport through the BBB by assessing the transport of 10 exosome populations derived from normal or malignant mouse or human cell culture supernatants in an in vivo mouse model (PMID: 32575812). The results demonstrated that, although all EV populations were capable of crossing the BBB in vivo, the crossing rates varied by as much as tenfold. Besides mechanistic differences, the transport of some EV populations was dependent on the mannose 6‐phosphate receptor, while other EVs were transported via adsorptive transcytosis. Furthermore, LPS activation of the innate immune system increased the uptake of six exosome populations but decreased the passage of one EV sample. Findings of Matsumoto and colleagues also support the effect of inflammation on the BBB crossing ability of EVs (Matsumoto et al. 2017). They demonstrated that aSyn‐containing EVs derived from red blood cells of Parkinson's disease (PD) patients were able to cross the BBB both in an in vivo mouse and in an in vitro brain microvascular endothelial cell (BMEC) monolayer transwell model. LPS administration and the subsequent increase in BBB permeability resulted in enhanced crossing ability of the vesicles both in vivo and in vitro. Based on these results, the authors postulated that systemic inflammation occurring in humans might increase the efflux of aSyn‐containing EVs in the CNS and thus contribute to the pathomechanisms of PD. The content of vesicles also can be a key factor in determining the trans‐BBB passage of EVs. In vitro and in vivo experiments conducted with cancer cells revealed that EVs derived from brain metastatic cancer cells provoked the destruction of the BBB, leading to enhanced extravasation and metastasis formation of the cancer cells (Tominaga et al. 2015). The microRNA miR‐181c, found within the EV, was identified as a key player in this process. Through down‐regulation of its target gene, PDPK1, miR‐181c induces abnormal localization of the cytoskeletal protein actin.

The ability of EVs to exit the CNS is evidenced by the presence of EVs of neuronal, astrocytic, and oligodendrocyte origin in the peripheral blood (for more detailed information, see e.g., Kluge et al. 2022; Hornung et al. 2020). A commonly used approach for the enrichment of specific subtypes of the vesicles is via immunoprecipitation with the use of antibodies targeting membrane markers characteristic of the aforementioned neural cell populations. However, as the cellular markers are often not exclusive to one or another cell type, and since EVs of CNS origin are present in very low abundance in the blood, the selection and further validation of the successful isolation of specific types of vesicles pose great challenges. Nonetheless, efforts made to overcome these difficulties are greatly beneficial, since analysis of the cargo of CNS‐derived EVs using peripheral biofluids could give insight into the status of the CNS. Thus, the use of EVs might pave the way to the identification of long‐awaited, easily accessible biomarkers for neurological disorders such as neurodegenerative diseases, which are currently unavailable or rely on more complex procedures such as lumbar puncture.

### 4.3 HEV at the Blood‐Urine Barrier

Each kidney is composed of around one million nephrons, each built of a glomerulus and a tubular system. The glomerulus is the functional unit of the kidney where the urine is filtered from the blood. This happens at the glomerular filtration barrier (GFB, Figure 4C), which is composed of glomerular endothelial cells, glomerular basement membrane and podocytes. Filtration at the GFB is size and charge selective. Endothelial cell fenestrae have a size of 70–90 nm, while their glycocalyx further reduces effective pore size (Finch et al. 2023). The glomerular basement membrane, which is a gel‐like structure, allows the passage of particles of 6–8 nm in size (Du et al. 2017). Slit diaphragms of podocytes restrict passage of molecules larger than 4–11 nm. The combined size restriction of these three layers limits filtration of particles larger than 6–8 nm in hydrodynamic diameter (Huang and Gretz 2017). Thus, in physiological conditions, systemic EVs and OMVs are mainly retained from filtration but can act on glomerular endothelial cells, thereby mediating systemic signals to the kidney (Figure 4C). In addition to the cells that directly build the GFB, mesangial cells within the glomerulus can regulate the capillary surface filtration area and influence barrier function by secretion of growth factors and cytokines (Ebefors et al. 2021). Usually, a very small fraction of urinary EVs originate from the circulation or kidney‐infiltrating cells (Pisitkun et al. 2004). However, systemic EVs could cross podocytes via transcytosis and are lost from the circulation in glomerular disease when the filtration barrier gets leaky. Furthermore, there is evidence for an intraglomerular crosstalk mediated by EV. EV found in the urine can mediate interorgan signalling but also crosstalk between glomerular and tubular cells and between different segments of the tubular system. However, most data on this interaction arise from ex vivo experiments in cell culture or mice (Jeon et al. 2020; Munkonda et al. 2018).

EVs that are released into the circulation by other organs might play a role in interorgan crosstalk, including the kidney. Due to the size selectivity of the GFB, these EVs mainly target the endothelial side of the barrier, where they can contribute to diseases. For example, in preeclampsia, a disease in pregnancy, placenta‐derived EVs carrying anti‐angiogenic factors might lead to glomerular endothelial cell dysfunction and proteinuria, which are hallmarks of this disease (Gebara et al. 2021). Circulating EV might also contribute to renal damage in autoimmune diseases, such as systemic lupus erythematosus, antiphospholipid syndrome and ANCA‐associated disease (Mazzariol et al. 2021). Moreover, EV circulating in the blood could mediate organ damage in cardio‐renal diseases (Chatterjee et al. 2023; Gabisonia et al. 2022). In vitro studies suggest that altered EVs mediate communication between glomerular endothelial cells and podocytes, leading to podocyte dysfunctions. Glucose‐stimulated glomerular endothelial cells release EV enriched with transforming growth factor β1 and can induce epithelial‐mesenchymal transition and podocyte damage (Wu et al. 2017). Furthermore, glomerular mesangial cell‐derived EVs were reported to modulate the renin‐angiotensin system and to stimulate fibronectin synthesis (da Silva Novaes et al. 2019). In the future, it will be interesting to see whether these ex vivo findings also hold up in in vivo models. Podocytes, positioned at the urinary space, release EV, which can be taken up by any other cell type of the subsequent urine secretion system. As a result, EV released from podocytes can mediate the transition from glomerular injury to tubular fibrosis. When podocytes are exposed to stress stimuli, they release an elevated quantity of EVs in mice, which are then internalized by proximal tubule cells and promote a profibrotic phenotype in vitro (Burger et al. 2014).

### 4.4 HEV at the Blood‐Placenta Barrier

The blood‐placenta barrier separates the growing foetus from the maternal system (Figure 4D). The placenta supplies the foetus with oxygen and nutrients and at the same time protects it from toxins, pathogens and maternal diseases. Additionally, the blood‐placenta barrier enables tolerance between the maternal and embryonic immune systems. To specifically fulfil these different functional properties, a large surface area and a specialized blood‐placenta barrier separating maternal and foetal circulation are required (Burton and Fowden 2015). The chorionic villi represent the functional unit of the blood‐placental barrier, which is formed by foetal vascular endothelial cells and three different types of trophoblast cells separating maternal and foetal blood spaces (Hernandez‐Verdun 1974; Enders 1965). The outer monolayer of sinusoidal trophoblast giant cells (S‐TGCs) lines the maternal blood sinusoids, and the two inner layers of syncytiotrophoblasts (syncytiotrophoblast‐macrophage I and II, STBI and STBII) cover the foetal endothelium (Simmons et al. 2007; Watson and Cross 2005). S‐TGCs have mainly endocrine functions (Simmons et al. 2007), being loosely attached to the underlying syncytial layers through desmosomal adhesions, providing STB‐I cells direct access to the maternal blood through highly permeable fenestrations (Hernandez‐Verdun 1974; Coan et al. 2005; Davies and Glasser 1968). The syncytiotrophoblast cell layers are a true multinucleated syncytium, tightly adhered to each other through tight junctions, situated on basement membranes overlying the foetal capillary endothelium (Hernandez‐Verdun 1974; Enders 1965; Coan et al. 2005; Jollie 1964). This firm arrangement functions as a barrier against the maternal circulation by preventing lateral and paracellular diffusion of substrates and preventing the vertical transmission of pathogens from the maternal blood (Robbins et al. 2010; Burton and Watson 1997). Exchange across the blood‐placental barrier takes place through diffusion, transporter‐mediated mechanisms, and endocytosis/exocytosis (Burton and Fowden 2015). To facilitate maternal‐foetal exchanges, both STB layers express specific transporters such as GLUT 1, GLUT 3 and monocarboxylate transporter (MCT) (Nagai et al. 2010; Illsley 2000; Takata and Hirano 1997). Transport through endo‐ and exocytosis has been described for IgG, transferrin‐bound iron, and low‐density lipoprotein (LDL) (Lencer and Blumberg 2005; Claypool et al. 2004; Dickinson et al. 1999; Pearse 1982).

The syncytiotrophoblast is of foetal origin. EV released by the syncytiotrophoblast are therefore often described as foetal EV. However, several authors describe them as placental EV. Nevertheless, bidirectional transfer across the placental‐blood barrier has been demonstrated for maternal and placental exosomes as well as for exosomes originating directly from the foetus (Sheller‐Miller, Choi, et al. 2019). Emerging data suggest that there are mechanisms for systemic transport of placenta/foetal‐derived EV into the maternal system, including the maternal lungs and the maternal liver (Kang et al. 2023). Further, OMV from the mother's microbiota communicate with the foetus, as they cross the placental barrier and thereby influence the foetal system (Kaisanlahti et al. 2023; Chen et al. 2023).

During pregnancy, the placenta, as part of the foetus, continuously releases EV of a large size range into the maternal circulation. This includes extremely large EV (approximately 70 µm in diameter; termed macro‐EV), but also EV of medium (100–1000 nm) and smaller size (30–150 nm) (Lapaire et al. 2007). Placental foetal EV originate from the syncytiotrophoblast. Placental maternal EV stem from villous cytotrophoblasts and endovascular trophoblasts located in the maternal spiral arteries (Tong and Chamley 2015). Placenta‐derived EV are characterized through their positivity for the STB marker placental alkaline phosphatase (PLAP) and can be detected in the maternal blood starting at the 6th week of gestation, with their quantity progressively rising as pregnancy advances (Sarker et al. 2014; Salomon, Torres, et al. 2014).

In the physiological state of a normal pregnancy, HEV and particularly exosomes have been identified as important components of the feto‐maternal communication system during implantation and placentation (Mishra et al. 2021; Andronico et al. 2019), modulating maternal immune response (Mincheva‐Nilsson and Baranov 2014) promoting foetal vasculogenesis and maternal vascular adaptation (Jia et al. 2018; Salomon, Yee, et al. 2014). Moreover, they contribute to the preparation of the uterus for delivery (Sheller‐Miller, Trivedi, et al. 2019). Previous studies describe an association between the levels of circulating maternal placental and foetal placental EV and their contents with gestational complications, including gestational diabetes and preeclampsia (Salomon et al. 2017, 2016). STB‐derived exosomes have antiviral properties to protect the developing foetus (Bayer et al. 2016). Furthermore, changes in the miRNA‐profile of maternal and placental‐derived exosomes from pregnant women with foetuses that display Trisomy 21, congenital heart, or neural tube defects compared to women carrying healthy foetuses (Condrat et al. 2021; Erturk et al. 2016). Regarding BEV, recent studies suggest that microbiota‐derived EV serve as an interaction mechanism between maternal microbiota and the developing foetus, potentially playing a pivotal role in priming the prenatal immune system for gut colonization after birth (Turunen et al. 2023). Using a mouse model system, Kohli and colleagues showed that EV from cultured mouse endothelial cells led to placental inflammasome activation and structural changes consistent with placental malperfusion when administered to pregnant mice, suggesting that HEV may also carry pathogens from the mother to the placenta to induce changes in barrier integrity (Kohli et al. 2016).

In summary, successful pregnancy requires extensive exchange between the mother and the foetus. Bidirectional transfer of EV between the maternal and foetal systems plays an important role in this maternal‐foetal crosstalk. Hereby, EVs are pivotal for many physiological processes like implantation but also for pathological situations like preeclampsia. Nevertheless, there are many aspects in this area of EV research with multiple questions remaining unanswered. For example, the exact mechanisms by which placental EVs interact with the target cells and alter their properties are still unclear. Furthermore, it is not yet sufficiently investigated whether EVs are merely promoters or initiators of physiological or pathophysiological processes. In addition, there is a lack of larger clinical studies to capture the potential of EV as biomarkers in gestation‐associated diseases and to enable the step from bench to bedside.

### 4.5 HEV at the Synovial Barrier

The synovium is the barrier that encapsulates the diarthrodial joints and maintains the immune privilege of the joints. It has several other functions, including the production of viscous synovial fluid (SF), which is essential for joint mobility and mechanical cushioning. These multiple functions require a high degree of compartmentalization, and two main compartments can be distinguished: the synovial lining layer (LL), where macrophages monitor the immunological barrier and fibroblasts secrete and maintain the SF, and the stromal synovial sublining layer (SL), which is interspersed with supplying blood vessels and adipocytes that absorb mechanical loads. Representing a distinctive barrier within the joint, the synovial LL is composed of three different major cell types, including fibroblasts, macrophages, and endothelial cells (Culemann et al. 2019) (Figure 4E). Despite the absence of a true basement membrane, the fibroblasts within the LL express integrin α6 (CD49f), a marker for basement membrane anchoring (Rauber et al. 2024).

The synovial barrier is disrupted in inflammatory joint diseases, such as rheumatoid arthritis (RA) (Marsh et al. 2021), but also in degenerative articular diseases such as osteoarthritis (OA) (Mathiessen and Conaghan 2017). Barrier breakdown in RA is caused by several mechanisms, including proliferation of LL fibroblasts, infiltration of different immune cell types and the proliferation of endothelial cells, leading to loss of compartmentalization. An imbalance in the integrity of the articular cartilage resulting from the degradation of the extracellular matrix (ECM) predominantly underlies the pathogenesis of OA. This degradation is largely mediated by chondrocytes and fibroblasts via the secretion of metalloproteinases (MMP) (Grillet et al. 2023; Lories 2008). Emerging evidence underscores the multiple roles that EV play in regulating the synovial barrier in inflammatory and degenerative joint diseases. Numerous studies have demonstrated a significant increase in the abundance of EV in synovial fluid (SF), correlating with disease activity in both RA and OA (Foers et al. 2020; Distler et al. 2005; Berckmans et al. 2002). Furthermore, the origin and cargo of SF‐EV suggest a strong involvement in local immunopathology and may serve as a diagnostic tool for disease severity and treatment response (Boukouris and Mathivanan 2015). Previous studies show that SF‐EV in RA are mainly derived from platelets, neutrophils, T cells and monocytes. SF‐EV acts on fibroblasts and macrophages in the synovial LL as drivers of inflammation, but also in a protective manner: in RA, SF is notably enriched with CD66b+ AnxA1+ EV derived from neutrophils, as documented by Gyorgy et al. (2012) and Headland et al. (2015). These neutrophil‐derived EVs suppress inflammatory activation by directly targeting macrophages within the synovial barrier (Rhys et al. 2018). Furthermore, they exhibit protective effects against cartilage degradation by activating key anabolic genes in chondrocytes (Headland et al. 2015). Conversely, CD14+ monocytes and CD3+ T‐cell‐derived EVs, also abundant in SF, contribute to a proinflammatory milieu by inducing pro‐inflammatory metalloproteinases (MMP)‐1, 3, 9 and 13 as well as prostaglandin E2 through the upregulation of cyclo‐oxygenase 2 (COX‐2) in LL fibroblasts (Distler et al. 2005; Jungel et al. 2007). Platelet‐derived EV, notably CD41+ EV, which traverse the synovial barrier in RA, harbour proinflammatory proteins that activate LL fibroblasts and neutrophils upon internalization, as elucidated by Boilard et al. (2010) and Duchez et al. (2015). Additionally, these EVs contribute to local hypercoagulation and fibrin deposition, as highlighted by Berckmans et al. (2002). LL fibroblasts, primarily identified as recipients of SF‐EV, are also prolific producers of EV (LLF EV) themselves. LLF‐EVs play a pivotal role in promoting angiogenesis within the RA synovium by modulating p53/mTOR signalling in endothelial cells, as elucidated by Chen et al. (2022). Furthermore, LLF‐EV carries miR‐424, which disrupts the balance between pro‐inflammatory IL‐17‐expressing T helper cells (Th17) and regulatory T cells (Treg) by downregulating Foxp3 expression, a critical gene in Treg function, as revealed by Ding et al. (2020). As the breakdown of ECM in the articular cartilage is a key driver of disease severity in OA, recent but limited studies focus on elucidating the intricate roles of chondrocyte‐ and fibroblast‐derived SF‐EV: chondrocyte‐derived EV serve as a crucial messenger in the communication network with fibroblasts, regulating disease progression. This OA‐shaping response involves the upregulation of key mediators such as MMP‐13, COX‐2 and tumour necrosis factor alpha (TNFα) upon interleukin (IL)‐1β stimulation and, furthermore, a downregulation of Type II collagen expression, a crucial component of ECM homeostasis (Kato et al. 2014). Simultaneously, IL‐1β‐stimulated synovial fibroblasts demonstrate a notable propensity to release EV, subsequently promoting angiogenesis, another driver for disease progression in OA (Kato et al. 2014).

In summary, the synovium is a unique joint barrier that undergoes several remodelling processes in autoinflammatory and degenerative joint diseases such as RA and OA. Recent research shows a strong involvement of SF‐EV. SF‐EVs are important mediators in the cell‐to‐cell communication in the synovial barrier and carry pro‐ and anti‐inflammatory cargo. Therefore, SF‐EV offers a completely new potential to control the disease severity in inflammatory and degenerative joint diseases.

### 4.6 Interplay Between OMV and HEV

In the preceding section, we delved into the interaction of OMV and HEV with different barriers. Intriguingly, these two systems appear to influence each other in a highly dynamic and multifaceted manner. While the exact mechanisms of HEV impacting OMV release remain elusive, several studies have indicated that specific OMV or bacterial infections can directly influence the release of HEV from host cells. For instance, cells infected with *Helicobacter pylori* release a higher quantity of HEV compared to non‐infected control cells. However, whether this effect necessitates direct contact of host cells with *Helicobacter pylori* or if *Helicobacter pylori*‐derived BEV alone are sufficient remains uncertain (Qiang et al. 2022). Nevertheless, it has been demonstrated that both, *Helicobacter pylori*‐derived OMV (or other EV species) and host‐derived HEV from *H. pylori*‐infected cells contribute to shaping the immunological response to the disease. Specifically, OMV possesses pro‐inflammatory and immunosuppressive properties, facilitating *Helicobacter pylori* evasion from the host's immune system and promoting cell infection (Wang et al. 2023). Another example of the interplay between OMV and HEV is the intraperitoneal application of OMV, which induces sepsis in mice, characterized by symptoms such as hypothermia, cytokine storm, and ocular exudate. Interestingly, this sepsis phenotype can be mitigated through the administration of EV derived from mesenchymal stem cells (MSC). These MSC‐derived EVs exhibit systemic distribution in mice similar to that seen for the OMVs and elicit the release of interleukin‐10 (IL‐10) as a protective factor (Park et al. 2019). Collectively, recent research has highlighted the potential for crosstalk between HEV and OMV in various physiological and pathological contexts. While the interplay between both EV populations represents a fascinating area of research with far‐reaching implications for microbiology, immunology, and translational medicine, molecular mechanisms and functional consequences are poorly understood.

## 5 Current Challenges in OMV and HEV Research

To explore the intricate relationship between EVs and their mechanistic functions, their application as diagnostic markers or therapeutic agents arises from various factors. These include the diverse surface and cargo compositions of OMV and HEV, dynamic changes in their composition during interactions with different cells or environments and technical limitations. Interestingly, both OMV and HEV challenge the research field with similar problems stemming from their physical characteristics, diversity in cargo and complexity in patient‐derived samples.

### 5.1 Diverse Surface and Cargo Compositions

EVs are carriers of various surface and cargo molecules, including proteins, lipids, nucleic acids, sugars and metabolites (Harmati et al. 2021; Zaborowski et al. 2015). Regarding BEV, various components such as lipids, LPS, outer membrane proteins, and encapsulated periplasmic content have been identified (Sartorio et al. 2021). A number of studies have demonstrated that both the cargo contents and surface compositions of EV impact barriers, for instance barrier permeability. HEV originating from patient‐derived glioblastoma cells or present in sera of patients have been shown to contain high levels of Semaphorin 3A, a pro‐permeable factor, on their surface, linked to the loss of endothelial barrier integrity (Treps et al. 2016). BEV derived from the enteric pathogen *Campylobacter jejuni* have demonstrated proteolytic activity that cleaves junction proteins such as E‐cadherin and occludin, suggesting the distinct role of BEV in modulating the permeability of the intestinal barrier (Elmi et al. 2016). Extensive efforts have been devoted to profiling EV composition in correlation with specific health and pathological conditions, employing techniques such as proteomics (Charest 2020), RNA sequencing (Turchinovich et al. 2019), lipidomics (Perpina‐Clerigues et al. 2023; Sun et al. 2022), and, more recently, metabolomics (Sun et al. 2022). These endeavours hold great potential for advancing the development of biomarkers and enhancing our understanding of the distinct functions of EV. However, the diversity of EV surface and cargo composition complicates determination of the functional contributions of individual components. It is plausible that the functions of EV likely involve the synergistic effects of multiple molecules. For instance, studies on the protein interaction network of HEV derived from human colorectal cancer cells have revealed influences on protein sorting during EV formation and pathophysiological functions (Choi et al. 2012). Functional studies examining the impact of the protein/lipid ratio on EV rigidity, circulation stability, and interaction with recipient cells (Record et al. 2018) further underscore the importance of considering EV functions as dynamic entities, a facet less addressed in past studies. Future research should not only isolate individual components but also consider their function as a cohesive whole. Validation of potential markers through multi‐omics analyses is imperative for understanding the crosstalk of EV and barriers. Additionally, employing systems biology approaches to analyse the intricate network of interactions between different components within EV, including biomolecule interaction networks within EV, signalling pathways, and regulatory networks in intercellular communication, will provide a more holistic understanding of the interdependent relationship of EV and barriers in (patho)physiological processes.

### 5.2 Dynamic Changes of EV During the Interaction With Barriers

Another challenge is linked to the dynamic of EVs and their changing composition during the interaction with barriers. Both HEV and BEV have demonstrated the capability to traverse various barriers through paracellular (between cells) and/or transcellular pathways (through cells, transcytosis) (Chatterjee, Yang, Ma, Wu, et al. 2020; Stentz et al. 2018). Regarding transcytosis, diverse endocytic pathways have been suggested, including clathrin‐mediated endocytosis, caveolae‐mediated endocytosis, and clathrin‐ and caveolae‐independent endocytosis (Stentz et al. 2018; Ramos‐Zaldivar et al. 2022). However, the investigation of endocytic pathways often involves the use of chemical endocytosis inhibitors (Stentz et al. 2018; Ramos‐Zaldivar et al. 2022) that are not very specific (Dutta and Donaldson 2012), leading to an unclear understanding of the underlying mechanisms. It is probable that multiple endocytic pathways are implicated, further complicating this matter. The destiny of EV, whether internalized or directed through the paracellular and transcytosis pathways, holds significance in comprehending their function at barriers and post‐barrier passage. Investigating the underlying mechanisms becomes pivotal, particularly when considering the use of EVs as delivery vehicles for therapeutic purposes. Once internalized in barrier cells, EVs encounter diverse fates. The predominant intracellular transport mechanism involves the fusion of internalized EVs with early endosomes, subsequent transformation of early endosomes into late endosomes, and ultimately fusion with lysosomes for degradation (Stentz et al. 2018). However, based on variations in membrane structures and endocytic routes of EV uptake, EVs can also bypass the endosomal and lysosomal pathway, gaining release through, for instance, transport via the endoplasmic reticulum/Golgi complexes (Stentz et al. 2018; Qiu et al. 2023). This intricate interplay between EV and barrier cells poses a substantial challenge, demanding a deeper understanding of the multifaceted processes involved. Moreover, a research aspect that remains under‐represented is the dynamic changes in surface compositions of EV during their interaction with barriers. Due to their relatively large surface‐to‐volume ratio, EVs engage in crucial surface interactions with surrounding cells and extracellular matrix molecules, potentially manifesting their roles as signalling factors (Buzás et al. 2018). For instance, studies have demonstrated that a significant proportion of exosomes co‐isolate with albumin (Baranyai et al. 2015) and other plasma proteins, as well as lipoproteins (Buzás et al. 2018). Hence, it is plausible that EVs undergo alterations in their surface composition when interacting with barriers. A comprehensive understanding of these interactions is vital for unravelling the fates, distribution, and functions of EV. Unfortunately, limited studies have addressed this aspect, likely attributed to technical constraints in tracking dynamic composition changes and employing appropriate barrier models. Overcoming these challenges could be achieved through a combination of enhanced purification methods, advanced imaging approaches to track individual EVs, the development of barrier models, and integrated omics analysis of surface components.

### 5.3 Technical Challenges

EVs exhibit remarkable heterogeneity in their structure, size and compositional features. Beyond this diversity, the challenges intensify due to the constraints in available quantities when working with EV derived from cell, animal and patient materials. These factors collectively constrain the techniques employed for the purification and characterization of EV. Various approaches have been developed to enrich EV from different sources. Classical techniques, such as differential centrifugation, ultracentrifugation, density gradient centrifugation, and size exclusion chromatography, leverage the biochemical and biophysical features of EV, while immune‐isolation involves using specific markers for targeted surface molecules (Harmati et al. 2021; Ramirez et al. 2018). However, each method comes with its own set of advantages and disadvantages (Ramirez et al. 2018; Davidson et al. 2023; Liangsupree et al. 2021). Combining these conventional methods has proven effective in enriching different EVs from a single biological sample, as exemplified by the separation of both HEV and BEV from a single blood or faecal sample using a combination of differential centrifugation, density gradient centrifugation, and size exclusion chromatography (Tulkens, De Wever, et al. 2020). Recent developments have introduced microfluidics‐based on‐a‐chip systems for EV purification, relying on immunoaffinity capture targeting specific surface markers or separating particles based on biophysical properties like size, density, and compressibility. These microfluidic approaches offer advantages such as low sample volumes, high throughput, and high reproducibility (Guo et al. 2018). Given the highly heterogeneous characteristics of different EVs, it is difficult to develop universal solutions. Advances in characterizing individual EV types hold the potential to design tailored microfluidic systems for future studies. In addition to the challenge of purifying EV, another technical hurdle relates to detecting them. A specific example pertains to the detection of OMV. To detect OMV in a complex biofluid such as human plasma, factors such as OMV titres during infection and the sensitivity and selectivity of the detection device must be taken into account. Low titres of OMV and/or insufficient sensitivity of the detection device (e.g., weak antibody binding to the antigenic OMV target) could result in a weak positive signal, and high titres of extraneous EV or poor selectivity of the detection device (e.g., nonselective antibody binding to non‐OMV targets) may lead to false‐negative results. Therefore, an initial purification and/or concentration step of the OMV could enhance both sensitivity and selectivity in detection.

## 6 Translational Research Approaches to Integrate OMV and HEV Into Clinical Application and Diagnostics

### 6.1 OMV in Clinical Application and Biomarker Development

As introduced above, BEVs are considered promising targets and tools for vaccination. The ability of BEV to stimulate antigen presentation and to foster the development of memory T cells has attracted interest in using OMV in vaccine development against many infectious agents. Two characteristics make BEV particularly interesting: (1) OMV are non‐replicating spherical nanostructures that contain pathogen‐associated molecular patterns (PAMPs) as well as bacterial surface antigens and thus, activate innate and adaptive immune responses. (2) BEVs are ideally sized for uptake by immune cells. Thus, BEVs are promising targets for vaccination against many microbial pathogens because of their immunomodulatory, antigenic and adjuvant properties. Importantly, to vary their antigen concentrations and structures, to reduce their endotoxicity or to load them with homologous, heterologous and even non‐microbial epitopes, BEV can be bioengineered and utilized for the treatment of non‐infectious diseases. Because BEV can display multiple tumour antigens and neoantigens, they could also enable a personalized treatment of cancer patients (Cheng et al. 2021). A detailed discussion on these BEV‐based cancer vaccine avenues is nevertheless beyond the scope of the current work but is reviewed elsewhere (Masforrol et al. 2017). Overall, besides vaccination and as a vehicle for infection control, BEVs represent promising tools for immunotherapy and drug delivery, particularly in cancer patients.

Based on the nature of the targeted pathogen, BEV can be delivered by various administration routes, such as nasal, intratracheal and oral applications. Most existing adjuvants fail to deliver antigens to mucosal tissues; in contrast, bacterial BEVs are promising mucosal vaccine candidates because they interact with or enter mucosal epithelial cells, modulate subsequent mucosal immune responses of the host and overcome this hurdle (Kaparakis‐Liaskos and Ferrero 2015; Charpentier et al. 2023). Indeed, BEV‐based vaccines against many mucosal pathogens, including *E. coli*, *Salmonella*, *Yersinia, Vibrio* and *Campylobacter* spp., *Helicobacter pylori* and *Acinetobacter baumannii*, as well as *Neisseria* (*N*.), *Pneumococcus* and *Shigella* spp., have been intensively studied (Majumder et al. 2023; Singh et al. 2023; Bjanes et al. 2023; Zanella et al. 2021; Gerritzen, Salverda, et al. 2019; Gerritzen, Martens, et al. 2019; Lee et al. 2018; Adriani et al. 2018; Petousis‐Harris 2018). Importantly, there are already two licensed BEV vaccines against invasive *N. meningitidis* serogroup B available. Interestingly, meningococcal group B‐derived BEV, containing the antigen collection and pharmaceutical ingredients of both licensed vaccines, might even protect against *N. meningitidis* serogroup C or the related *Neisseria* species *N. gonorrhoeae* (Masforrol et al. 2017; Petousis‐Harris 2018). Another interesting experimental example is the assessment of pneumococcal BEV that were able to induce cross‐protection in mice, which was not dependent on pneumolysin (Narciso et al. 2022). In any case, the efficacy of BEV‐based vaccines also strongly depends on the design of an appropriate delivery formulation, such as incorporation into biodegradable microparticles (Mehanny et al. 2022).

Different viruses can be targeted by bacterial‐derived BEVs, including the Middle East Respiratory syndrome–related coronavirus (MERS‐CoV), influenza virus or severe acute respiratory syndrome coronavirus type 2 (SARS‐CoV‐2). While the receptor‐binding domain (RBD) or the spike (S) protein are embedded as vaccination targets against both coronaviruses into *E. coli* or *N. meningitidis* BEV, H1 hemagglutinin or M2e antigen serve as targets for vaccination against influenza (Lieberman 2022; Kashyap et al. 2022). Interestingly, immunization with BEV displaying a chimeric fusion protein of MERS‐CoV‐2 RBD and influenza hemagglutinin elicited neutralizing antibodies against both viral antigens (Shehata et al. 2019). The lack of critical posttranslational modifications, such as glycosylation, may limit the use of BEV endogenously loaded with complex viral antigens. In contrast, HEV can be engineered to incorporate fusion‐competent viral surface proteins suggesting presentation of viral glycoproteins in their natural conformation (Kuate et al. 2007; Temchura et al. 2008).

BEV can protect against parasites as well. For example, *Serratia ureilytica*, a bacterial gut commensal of mosquitos, delivers AmLip, an effector lipase, to Plasmodium parasites via BEV. After a blood meal, host serum induces *Serratia ureilytica* to release BEV and the antimalarial effector protein AmLip into the mosquito gut, leading to subsequent killing of malaria parasites (Gao et al. 2023). In addition, parasites commonly use BEV to interact with the host. EVs are also important for the crosstalk between gastrointestinal parasites and the gut microbiota of the host (Rooney et al. 2022). Since these EVs also contain immunomodulatory components, they might be promising targets for vaccination themselves (Alfandari et al. 2023).

In summary, BEVs are promising tools for (mucosal) vaccine design and an emerging and promising platform for vaccine development against bacteria, parasites, viruses and cancer. Their ability to replicate the immunomodulatory effects of bacteria positions them even as potential therapeutic tools. They also exhibit ideal adjuvant properties and innate humoral immune surveillance due to their inherent immunogenicity. Moreover, fusion of heterologous antigens to BEV proteins allows an expression of non‐native antigens on the BEV surface. An incorporation of tissue‐ and/or cell‐specific moieties into BEV through genetic or chemical modification even allows targeted drug delivery (Heinrich et al. 2023; Herrmann et al. 2021). Thus, BEVs are a promising platform for non‐regenerative and acellular vaccines and potentially for immunotherapy and drug delivery. In contrast, the consistency of yields, immunogenicity, toxicity, and loading of BEV are some of the challenges of this system. However, there is continuous improvement in upscaled extraction methods to increase vesicle yield and genetic modifications to avoid possible endotoxicity. Moreover, engineering technologies allow the synthesis of immunogenic bacterial vesicles and avoid the immunotoxicity of purified BEV (Park et al. 2023). By harnessing the versatility of OMV, we may unlock new avenues for safer and more targeted approaches to human healthcare, providing innovative solutions for a wide range of health challenges.

### 6.2 HEV in Clinical Application and Biomarker Development

In light of the important role of EVs in intercellular communication and their ability to cross different barriers of the human body, they can be considered as bona fide delivery systems (Massey et al. 2021). Consequently, utilizing either modified EVs to transport therapeutic agents or vesicles derived from specific cell types bearing characteristics of those cells seems a rational and well‐grounded therapeutic approach. Moreover, the ability of EVs to cross physiological barriers makes them promising candidates for biomarker research: analysis of vesicles derived from blood, urine or other easily accessible biofluids could offer the possibility to gain insight into the status of various organs without the need for biopsies and other invasive interventions.

**FIGURE 5:**
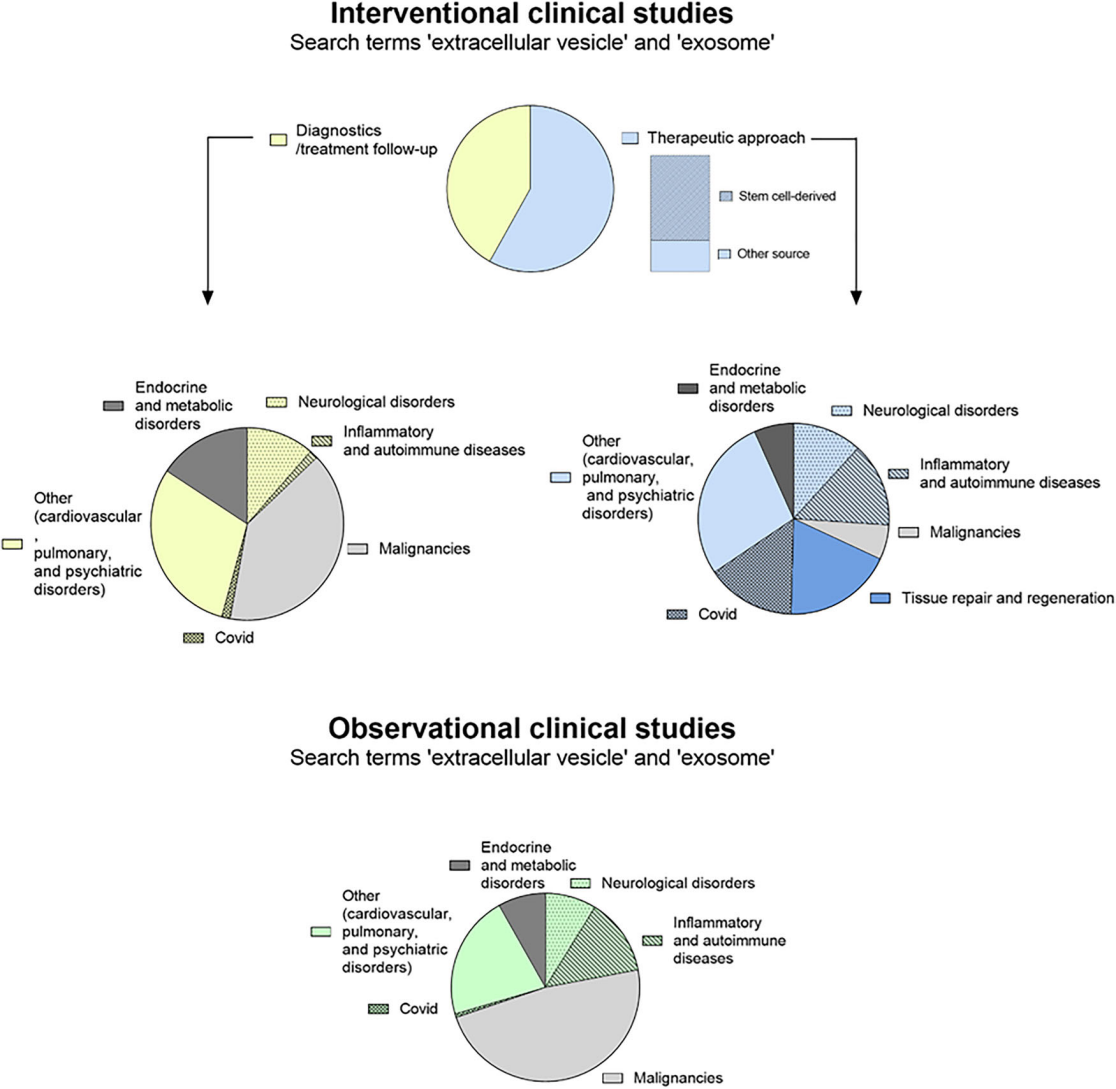
Pie‐chart diagram of current clinical studies involving EVs from the host system. A complete table covering the individual studies can be found as Table S1.

In light of these, intensive research is focusing on the development of therapeutic and diagnostic approaches utilizing EVs. This is well reflected by the number of relevant clinical trials listed on the website of The National Library of Medicine (NLM) (www.clinicaltrials.gov). Queries performed on 16 January 2025, using the search terms ‘extracellular vesicle’ or ‘exosome’ in the search category intervention/treatment resulted in a total of 348 clinical studies, out of which 189 belonged to interventional trials (Table S1). It is noteworthy to mention that within 1 year (from January 2024 to January 2025) the number of clinical trials listed in the registry showed an approximately 30% increase (263 to 348). The majority of these trials aim to utilize EVs as a therapeutic approach, mainly in inflammatory and autoimmune diseases, regenerative medicine, neurological and psychiatric disorders and other diseases affecting the cardiovascular and pulmonary system (Figure 5, Table S1). With respect to trials focusing on the potential of EVs in diagnostics and monitoring treatment effects, most of the studies focus on malignant diseases. The dominance of clinical trials conducted in the field of malignant diseases is also noticeable when analysing the search results of observational studies (Figure 5; Table S1).

Most of the trials involving EV‐based therapeutic approaches use stem cell‐derived vesicles, primarily those originating from mesenchymal stem cells (MSCs). The great potential attributed to MSC‐based therapy is reflected in the number of over 60 FDA‐approved clinical trials using these cells (but not EVs derived from those), mostly for the treatment of conditions requiring tissue healing, haematopoietic stem cell transplantation, and in cases targeting autoimmune diseases (Kou et al. 2022). Many of these trials attempt to exploit the immunomodulatory activity of MSCs, which exert effects on various cells of the adaptive as well as the innate immune system, resulting in an anti‐inflammatory milieu and facilitation of the survival of damaged cells (Uccelli et al. 2008). With regard to the therapeutic use of MSCs, serious concern is raised regarding their adverse effect in the promotion of metastatic spread and tumour growth, partially due to their pro‐angiogenic potential (Galiè et al. 2008; Karnoub et al. 2007). Therefore, great importance should be given to studies that have shown that the desired immunomodulatory effects of MSCs are not exclusively due to the engraftment of cells in target tissues but also due to the secretion of paracrine factors (Huang et al. 2014), such as EVs (Wiest and Zubair 2020; Cosenza et al. 2018; Lai et al. 2010). The realization that the vesicles harbour the therapeutic effects of the parent MSCs (Kou et al. 2022; Lai et al. 2010) prompted the assessment of the use of MSC‐derived EVs in the treatment of various human diseases (Table S1). A great advantage of cell‐free treatment strategies over using live‐cell‐based therapies is the elimination of safety concerns related to the adverse effects due to the self‐replicating and differentiation abilities of the cells or cell rejection by the immune system (Jafarinia et al. 2020). The use of MSC‐derived EVs, however, presents specific challenges. Among the main difficulties are the large‐scale production of clinical‐grade EVs together with controlling preparation purity and minimization of batch‐to‐batch differences (Rehman et al. 2023). These challenges can be mitigated with careful donor selection, development and implementation of standardized good manufacturing practices (GMPs), and by the strict control of the safety of preparations by the use of potency assays for specific disease conditions (Wiest and Zubair 2020). Given the great potential that MSC‐derived EVs hold, efforts in these directions should be encouraged. EVs are promising candidates to serve as delivery vehicles for therapeutic agents. Among the features making them suitable to fulfil this role are (1) their ability to cross the BBB, (2) their enhanced bioavailability in the gastrointestinal tract (Rehman et al. 2023), and (3) their capacity to remain in the circulation for an extended time due to their decreased immune‐activator properties as compared to their synthetic counterparts (e.g., liposomes) (Massey et al. 2021). Indeed, various agents have been successfully loaded into vesicles, including molecules for genetic therapies such as siRNAs and miRNAs and small‐molecule anti‐cancer drugs, namely cisplatin and doxorubicin (Massey et al. 2021). Moreover, a large protein, such as the antioxidant catalase, was also successfully loaded into vesicles, and transferred through the BBB to exert neuroprotective and anti‐inflammatory effects in a 6OHDA mouse model of PD, despite it being prone to deactivation and rapid degradation (Haney et al. 2015).

The use of EVs in relation to cancer has gained particular attention both in respect of therapeutic approaches and for diagnostic and treatment follow‐up purposes. The high proportion of clinical trials aiming to use EVs as a source of biomarkers in various cancer types well reflects this (Figure 5, Table S1). The majority of studies analyse EVs enriched from body fluids such as whole blood, plasma, serum, and urine, and aim at identifying EV‐derived signatures characteristic of the tumour. Several clinical trials have already been completed (ClinicalTrials.gov; Table S1). However, only a few have summarized the trial outcome in publicly available original research publications so far (Buscail et al. 2019; Huang et al. 2020; Kretschmer, Kajau, et al. 2022; Kretschmer, Tutrone, et al. 2022), while results of further studies are still eagerly awaited.

For diseases with manifestations in the urogenital system, EVs from urine samples hold the promise to become prime diagnostic markers. For example, podocyte‐derived podoplanin‐expressing urinary EVs were reported to be a marker for glomerular injury in diabetic mice, and urinary WT1‐expressing EVs were suggested as a non‐invasive biomarker for podocyte injury in focal segmental glomerulosclerosis (FSGS) and diabetic nephropathy (Delrue et al. 2023; Zhou et al. 2013). Moreover, urinary exosomal miR‐193a was suggested to be a novel non‐invasive biomarker for the diagnosis of primary FSGS—a disease affecting podocytes of the GFB (Huang et al. 2017). One of the biggest challenges in transforming urinary EVs into reliable clinical markers is the lack of standardized methods for urine collection, EV isolation and normalization. Furthermore, urine might be contaminated with microbiota, is variable in pH, osmolality, and concentration, and is influenced by certain medications and diet. To overcome this, the urinary task force within the *Rigor and Standardization Subcommittee* of ISEV has been formed (Erdbrügger et al. 2021)

## 7 Future Direction

To date, there is numerous data available showing how BEV and HEV interact with different human barriers. Nevertheless, there are also notable shortcomings in the research field that we should focus on within the coming years. First of all, there is a pressing need to identify standards for vesicle enrichment and characterization (De Langhe et al. 2024; Van Deun et al. 2017). A very useful step is the introduction of guidelines from ISEV (Welsh et al. 2024), and adherence to these guidelines is imperative. We must explore suitable methodologies that allow us to separate different subpopulations from vesicle preparations to gain a more comprehensive understanding of the composition, quality and quantity of specific vesicle subtypes (e.g., from a specific organ). Application of different vesicle preparations should focus on holistic approaches. We need to define standardized cell‐based barrier models that can be easily controlled and manipulated. It will be important to apply different omics techniques on the barrier and on the vesicle population to identify starting points for fundamental research approaches. Along the same line, we have to consider translational approaches that utilize in vivo or patient‐derived organoid models for more sophisticated research attempts. However, it will be important to have both, simple and complex models at hand to identify specific BEV‐ and/or HEV‐induced effects. Further approaches to translate certain BEV or HEV populations into biomarkers or intervention targets, premise insight into specific, vesicle‐mediated mechanisms that separate physiological from pathological states.

## 8 Conclusion

In this review, we have summarized the diverse and multimodal facets of BEV and HEV with a particular focus on mechanisms of barrier interaction and their transition into therapeutic agents and diagnostic markers. We hope that the fascinating field of EV research will grow further, resulting in a better understanding of the connection between bacterial and host‐derived EV. Both are two sides of the same coin, and holistic research approaches that consider both vesicle types are needed, especially in the context of complex pathologies such as neurodegeneration, cancer and immune‐mediated inflammatory diseases.

## Author Contributions

**Philipp Arnold**: visualization, writing–original draft Preparation, writing–review and editing. **Fanni Annamária Boros**: visualization, writing–original draft Preparation, writing–review and editing. **Jochen Mattner**: writing–original draft Preparation, writing–review and editing. **Gerald Seidel**: writing–original draft Preparation, writing–review and editing. **Chaofan Liu**: writing–review and editing. **Inga Victoria Hensel**: writing–review and editing. **Jan Van Deun**: writing–review and editing. **Raphael Schwendner**: writing–review and editing. **Janina Müller‐Deile**: writing–original draft Preparation, writing–review and editing. **Nina Sopel**: writing–original draft Preparation, writing–review and editing. **Andreas Ramming**: writing–original draft Preparation, writing–review and editing. **Mario R. Angeli**: writing–original draft Preparation, writing–review and editing. **Simon Rauber**: writing–original draft Preparation, writing–review and editing. **Cláudia Vilhena**: writing–review and editing. **Andreas Baur**: writing–review and editing. **Stefan Wirtz**: writing–review and editing. **Klaus Überla**: writing–review and editing. **Heiko Reutter**: writing–original draft Preparation, writing–review and editing. **Linda‐Marie Mulzer**: writing–original draft Preparation, writing–review and editing. **Alina C. Hilger**: writing–original draft Preparation, writing–review and editing. **Friederike Zunke**: writing–original draft Preparation, writing–review and editing. **Wei Xiang**: writing–original draft Preparation, writing–review and editing. **Gregor Fuhrmann**: writing–original draft Preparation, writing–review and editing. **Claudia Günther**: visualization, writing–original draft Preparation, writing–review and editing.

## Conflicts of Interest

The authors declare no conflicts of interest.

## Supporting information




**Supplementary Table**: jex270093‐sup‐0001‐TableS1.pdf

## Data Availability

All authors had access to the study data and reviewed and approved the final manuscript.
