# Supplementary material for: Two Sides of the Same Coin—Mechanistic Insight, Diagnostic Application and Therapeutic Translation of Bacterial and Host‐Derived Extracellular Vesicles
Source: J Extracell Biol. 2025 Oct 29;4(11):e70093. doi: 10.1002/jex2.70093 (PMC12570047; doi:10.1002/jex2.70093)
Supplement: Supplementary file 1 — Supplementary Table: jex270093‐sup‐0001‐TableS1.pdf [file JEX2-4-e70093-s001.pdf]

Supplementary Table 1. Interventional and observational clinical trials involving the use/analysis of EVs

EVs used for therapeutic approaches (Interventional clinical trials)				
**Disease group**	**Condition**	**Origin of EVs**	**Study Title**	**NCT Number**
Covid	Covid19	Stem cell	Intravenous Infusion of CAP-1002 in Patients With COVID-19	NCT04623671
	Long COVID-19 Syndrome	Stem cell	Safety and Efficacy of Umbilical Cord Mesenchymal Stem Cell Exosomes in Treating Chronic Cough After COVID-19	NCT05808400
	Covid19	Stem cell	A Pilot Clinical Study on Inhalation of Mesenchymal Stem Cells Exosomes Treating Severe Novel Coronavirus Pneumonia	NCT04276987
	Covid19, pneumonia	COVID-19 Specific T Cell derived exosomes	COVID-19 Specific T Cell Derived Exosomes (CSTC-Exo)	NCT04389385
	Covid19	CD24 Overexpressing Exosomes	Safety and Efficacy of Exosomes Overexpressing CD24 in Two Doses for Patients with Moderate or Severe COVID-19	NCT04902183
	Covid19, ARDS	Stem cell	Zofin (Organicell Flow) for Patients With COVID-19	NCT04384445
	Covid19, ARDS	Stem cell	The Use of Exosomes for the Treatment of Acute Respiratory Distress Syndrome or Novel Coronavirus Pneumonia Caused by COVID-19	NCT04798716
	Covid19, Pneumonia	Stem cell	A Clinical Study on Safety and Effectiveness of Mesenchymal Stem Cell Exosomes for the Treatment of COVID-19.	NCT05787288
	Covid19	Stem cell	Efficacy and Safety of EXOSOME-MSC Therapy to Reduce Hyper-inflammation In Moderate COVID-19 Patients	NCT05216562
	Covid19	CD24 Overexpressing Exosomes	A Phase II Randomized, Double-blind, Placebo-controlled Study to Evaluate the Safety and Efficacy of Exosomes Overexpressing CD24 to Prevent Clinical Deterioration in Patients with Moderate or Severe COVID-19 Infection	NCT04969172
	Covid19, ARDS	Stem cell	Extracellular Vesicle Infusion Treatment for COVID-19 Associated ARDS	NCT04493242
	Covid19, Pneumonia	Stem cell	Safety and Efficiency of Method of Exosome Inhalation in COVID-19 Associated Pneumonia	NCT04602442
	Covid19, ARDS	Stem cell	Safety and Effectiveness of Placental Derived Exosomes and Umbilical Cord Mesenchymal Stem Cells in Moderate to Severe Acute Respiratory Distress Syndrome (ARDS) Associated with the Novel Corona Virus Infection (COVID-19)	NCT05387278
	Covid19, Pneumonia	Stem cell	Evaluation of Safety and Efficiency of Method of Exosome Inhalation in SARS-CoV-2 Associated Pneumonia.	NCT04491240
	Covid19	Zofin (derived from amniotic fluid)	Zofin to Treat COVID-19 Long Haulers	NCT05228899
	Covid19	CD24 Overexpressing Exosomes	Evaluation of the Safety of CD24-Exosomes in Patients With COVID-19 Infection	NCT04747574
	Covid19, Postviral Syndrome, Dyspnea	Stem cell	ExoFlo Infusion for Post-Acute COVID-19 and Chronic Post-COVID-19 Syndrome	NCT05116761
	Covid19	Stem cell	Bone Marrow Mesenchymal Stem Cell Derived Extracellular Vesicles Infusion Treatment for Mild-to-Moderate COVID-19: A Phase II Clinical Trial	NCT05125562
Endocrine and metabolic disorders	Diabetes Mellitus Type 1	Stem cell	Effect of Microvesicles and Exosomes Therapy on Î²-cell Mass in Type I Diabetes Mellitus (T1DM)	NCT02138331
	Polycystic Ovary Syndrome	Plant	Plant Exosomes and Patients Diagnosed with Polycystic Ovary Syndrome (PCOS) 17	NCT03493984
	Familial Hypercholesterolemia	Stem cell	Exosome-based Nanoplatform for Ldlr mRNA Delivery in FH	NCT05043181
	Premature Ovarian Failure	Stem cell	Intra-ovarian Injection of MSC-EVs in Idiopathic Premature Ovarian Failure	NCT06202547
	Androgenetic Alopecia	Stem cell	Umbilical Cord-derived Mesenchymal Stem Cell Exosomes on Hair Growth in Patients with Androgenetic Alopecia	NCT06697080
	Androgenetic Alopecia	Stem cell	Exosome Treatment in Androgenetic Alopecia	NCT06539273

	Androgenetic Alopecia	Stem cell	Efficacy and Safety of AGE ZERO™ EXOSOMES to Treat Men and Women With Androgenetic Alopecia	NCT06482541
	Androgenetic Alopecia	Stem cell	Efficacy and Safety of Exosomes Versus Platelet Rich Plasma in Patients of Androgenetic Alopecia	NCT06239207
Tissue repair and regeneration	Diabetic ulcers	Stem cell	Evaluation of Personalized Nutritional Intervention on Wound Healing of Cutaneous Ulcers in Diabetics	NCT05243368
	Macular Holes	Stem cell	MSC-Exos Promote Healing of MHs	NCT03437759
	Anti-Aging	Stem cell	Mesenchymal Stem Cells Derived Exosomes in Skin Rejuvenation	NCT05813379
	Knee Injury	Stem cell	Clinical Efficacy of Exosome in Degenerative Meniscal Injury	NCT05261360
	Cutaneous ulcer	Plasma	Effect of Plasma Derived Exosomes on Cutaneous Wound Healing	NCT02565264
	Wounds and Injuries	Stem cell	Pilot Study of Human Adipose Tissue Derived Exosomes Promoting Wound Healing	NCT05475418
	Bone Loss	Stem cell	Autogenous Mesenchymal Stem Cell Culture-Derived Signalling Molecules as Enhancers of Bone Formation in Bone Grafting	NCT04998058
	Skin Grafting	Platelet	PEP on a Skin Graft Donor Site Wound	NCT04664738
	Burns	Stem cell	Safety of Extracellular Vesicles for Burn Wounds	NCT05078385
	Liver Failure	Stem cell	MSC-EV in Acute-on-Chronic Liver Failure After Liver Transplantation	NCT05881668
	Liver Failure	Stem cell	The Safety and Efficacy of MSC-EVs in Acute/Acute-on-Chronic Liver Failure	NCT05940610
	Chronic Otitis Media, Tympanic Membrane Perforation	Plasma	Use of Autologous Plasma Rich in Platelets and Extracellular Vesicles in the Surgical Treatment of Chronic Middle Ear Infections	NCT04761562
	Ulcer Venous	Serum	Autologous Serum-derived EV for Venous Trophic Lesions Not Responsive to Conventional Treatments	NCT04652531
	Segmental Fracture related Bone Loss	Stem cell	Treatment of Patients with Bone Tissue Defects Using Mesenchymal Stem Cells Enriched by Extracellular Vesicles	NCT05520125
	Apical Periodontitis	Stem cell	The Effect of Human Umbilical Cord Mesenchymal Stem Cells and Exosomes on the Healing of Postoperative Pain and Periapical Lesions in the Treatment of Apical Periodontitis: Randomized Controlled Clinical Study	NCT06764004
	Melasma	Stem cell	Umbilical Cord Mesenchymal Stem Cell-Derived Exosomes in the Treatment of Melasma	NCT06677931
	Mucositis (Chemotherapy- and Radiation-Induced)	Stem cell	Effects of Mesenchymal Stem Cell Supernatant on Prevention and Treatment of Skin/Mucosal Injury in Hematology Patients	NCT06599346
	Diabetic Foot Ulcer	Platelet (PEP; Purified Exosome Product)	Phase 2a Multi-Center Prospective, Randomized Trial to Evaluate the Safety & Efficacy of Topical PEP-TISSEEL for Diabetic Foot Ulcers (DFU)	NCT06319287
	Skin Regeneration Following Laser Surgery	Stem cell	Clinical Observation of Exosomes in Patients After Q-switched Laser Surgery	NCT06279039
	Melasma	Stem cell	Stem Cell Derived Exosomes in the Treatment of Melasma and Its Percutaneous Penetration	NCT06221787
	Impaired Glucose Tolerance	Stem cell	Preliminary Safety and Efficacy Study of Extracellular Vesicle Infusion in the Intervention of Age-related Phenotypes with Impaired Glucose Tolerance	NCT06495437
	Wound Healing	Stem cell	Randomized, Controlled, Multicenter Study of Extracellular Vesicles from Human Adipose Tissue Promoting Wound Healing	NCT06253975
Inflammatory and autoimmune conditions	Periodontitis	Stem cell	Evaluation of Adipose Derived Stem Cells Exo.in Treatment of Periodontitis	NCT04270006
	Atopic Dermatitis	Stem cell	Induced Pluripotent Stem Cell Derived Exosomes for the Treatment of Atopic Dermatitis	NCT05969717
	Dry Eye	Stem cell	Effect of UMSCs Derived Exosomes on Dry Eye in Patients With cGVHD	NCT04213248
	Perianal Fistula in Patients with Crohn's Disease	Stem cell	Safety of Injection of Placental Mesenchymal Stem Cell Derived Exosomes for Treatment of Resistant Perianal Fistula in Crohn's Patients	NCT05499156

	Knee Osteoarthritis	Stem cell	Intra-articular Injection of MSC-derived Exosomes in Knee Osteoarthritis (ExoOA-1)	NCT05060107
	Pulmonary infection	Stem cell	A Clinical Study of Mesenchymal Progenitor Cell Exosomes Nebulizer for the Treatment of Pulmonary Infection	NCT04544215
	Irritable Bowel Disease	Plant	Plant Exosomes +/- Curcumin to Abrogate Symptoms of Inflammatory Bowel Disease	NCT04879810
	KLH and Imiquimod Induced Skin Inflammation in Healthy Volunteers	EDP2939 (microbial EVs)	Evaluation of the Immunopharmacology of EDP1815 and EDP2939	NCT05682222
	Crohn´s Disease	Stem cell	Study of ExoFlo for the Treatment of Medically Refractory Crohn's Disease	NCT05130983
	Chronic Otitis Media related Temporal Bone inflammation	Plasma	Efficacy of Platelet- and Extracellular Vesicle-rich Plasma in Chronic Postsurgical Temporal Bone Inflammations	NCT04281901
	Ulcerative Colitis	Stem cell	Study of ExoFlo for the Treatment of Medically Refractory Ulcerative Colitis	NCT05176366
	Ulcerative Colitis, Inflammatory Bowel Disease	Whey protein milk	Whey Protein Milk-Derived Exosomes	NCT06755021
	IBD	Whey protein milk	WPMDE1 - Whey Protein Milk-Derived Exosomes Among Healthy Subjects	NCT06742203
	Knee Osteoarthritis	Stem cell	Mesenchymal Stem Cells Derived Exosomes in Osteoarthritis Patients	NCT06466850
	Knee Osteoarthritis	Platelet (PEP; Purified Exosome Product)	Phase 1b Clinical Trial to Evaluate PEP and EUFLEXXA for Knee Osteoarthritis (KOA)	NCT06463132
	Knee Osteoarthritis	Stem cell	Intra-articular Injection of UC-MSC Exosome in Knee Osteoarthritis	NCT06431152
	Retinitis Pigmentosa	Stem cell	Safety and Efficacy of Stem Cell Small Extracellular Vesicles in Patients with Retinitis Pigmentosa	NCT06242379
	Acute Respiratory Distress Syndrome	Stem cell	A Clinical Study of Mesenchymal Stem Cell Exosomes Nebulizer for the Treatment of ARDS	NCT04602104
	Acute Respiratory Distress Syndrome	O Exosomes Overexpressing CD24	Safety and Efficacy of EXO-CD24 in Preventing Clinical Deterioration in Patients with Mild-Moderate ARDS	NCT05947747
	Acute Respiratory Distress Syndrome	Stem cell	Extracellular Vesicle Treatment for Acute Respiratory Distress Syndrome (ARDS) (EXTINGUISH ARDS)	NCT05354141
	Psoriasis	Stem cell	Safety and Tolerability Study of MSC Exosome Ointment	NCT05523011
	Acute Respiratory Distress Syndrome	Stem cell	Bone Marrow Mesenchymal Stem Cell Derived Extracellular Vesicles Infusion Treatment for ARDS	NCT05127122
	Perianal Fistula due to Crohn's Disease	Stem cell	Study of ExoFlo for the Treatment of Perianal Fistulas	NCT05836883
	Severe Acute Respiratory Syndrome	Stem cell	Extracellular Vesicles from Mesenchymal Cells in the Treatment of Acute Respiratory Failure	NCT06002841
Malignancies	Head and Neck Cancer	Plant	Edible Plant Exosome Ability to Prevent Oral Mucositis Associated with Chemoradiation Treatment of Head and Neck Cancer	NCT01668849
	Advanced Hepatocellular Carcinoma (HCC), Gastric and Colorectal Cancer Metastatic to Liver	Not-specified	A Study of exoASO-STAT6 (CDK-004) in Patients with Advanced Hepatocellular Carcinoma (HCC) and Patients with Liver Metastases from EIther Primary Gastric Cancer or Colorectal Cancer (CRC)	NCT05375604
	Colon Cancer	Plant	Study Investigating the Ability of Plant Exosomes to Deliver Curcumin to Normal and Colon Cancer Tissue	NCT01294072
	Non- Small Cell Lung Cancer	Dendritic cells	Trial of a Vaccination with Tumor Antigen-loaded Dendritic Cell-derived Exosomes	NCT01159288
	Pancreatic cancer	Stem cell	iExosomes in Treating Participants with Metastatic Pancreas Cancer with KrasG12D Mutation	NCT03608631
	Rectal Cancer	Stem cell	Effects of Exosome Administration in Preventing Early Leakage in Rectal Cancer Patients Undergoing Low Anterior Resection	NCT06536712
	Acute Myeloid Leukemia	Stem cell	UCMSC-Exo for Chemotherapy-induced Myelosuppression in Acute Myeloid Leukemia	NCT06245746

Neurological disorders	Refractory Focal Epilepsy	Stem cell	Induced Pluripotent Stem Cell Derived Exosomes Nasal Drops for the Treatment of Refractory Focal Epilepsy	NCT05886205
	Alzheimer Disease	Stem cell	the Safety and the Efficacy Evaluation of Allogenic Adipose MSC-Exos in Patients with Alzheimer's Disease	NCT04388982
	Neuralgia	Stem cell	The Use of Exosomes in Craniofacial Neuralgia	NCT04202783
	Acute Ischemic Stroke	Stem cell	The Effect of GD-iExo-003 in Acute Ischemic Stroke	NCT06138210
	Premature Birth	Stem cell	The Pilot Experimental Study of the Neuroprotective Effects of Exosomes in Extremely Low Birth Weight Infants	NCT05490173
	Chronic Low Back Pain, Degenerative Disc Disease	Blood	Intra-discal Injection of Platelet-rich Plasma (PRP) Enriched with Exosomes in Chronic Low Back Pain	NCT04849429
	Post-stroke Dementia	Acupuncture-induced Exosomes	The Role of Acupuncture-induced Exosome in Treating Post-stroke Dementia	NCT05326724
	Cerebrovascular Disorders	Stem cell	Allogenic Mesenchymal Stem Cell Derived Exosome in Patients with Acute Ischemic Stroke	NCT03384433
	Muscular Dystrophies	Stem cell	A Study of CAP-1002 in Ambulatory and Non-Ambulatory Patients with Duchenne Muscular Dystrophy	NCT03406780
	Multiple System Atrophy - Parkinsonian Subtype (MSA-P)	Stem cell (Aleeto)	The Study of Safety and Preliminary Efficacy of Aleeto in Patients with MultIple System Atrophy	NCT06765733
	Amyotrophic Lateral Sclerosis	Stem cell	Exploring Nasal Drop Therapy with Small Extracellular Vesicles for ALS	NCT06598202
	Neuromyelitis Optica Spectrum Disorders	Stem cell	The Safety and Efficacy of NouvSoma001 in Neuromyelitis Optica Spectrum Disorders	NCT06620809
	Ischemic Stroke	Stem cell	The Safety and Efficacy of NouvSoma001 in Ischemic Stroke	NCT06612710
	Alzheimer Disease, Parkinson Disease, Lewy Body Dementia, Multiple System Atrophy, Fronto-temporal Dementia	Stem cell	HUC-MSC-sEV-001 Nasal Drops for Neurodegenerative Diseases	NCT06607900
Other	Healthy participants	Stem cell	A Tolerance Clinical Study on Aerosol Inhalation of Mesenchymal Stem Cells Exosomes in Healthy Volunteers	NCT04313647
	Multiple Organ Failure	Stem cell	Exosome of Mesenchymal Stem Cells for Multiple Organ Dysfuntion Syndrome After Surgical Repaire of Acute Type A Aortic Dissection	NCT04356300
	Retinitis Pigmentosa	Stem cell	The Effect of Stem Cells and Stem Cell Exosomes on Visual Functions in Patients with Retinitis Pigmentosa	NCT05413148
	Perianal Fistula	Stem cell	Safety and Efficacy of Injection of Human Placenta Mesenchymal Stem Cells Derived Exosomes for Treatment of Complex Anal Fistula	NCT05402748
	Decompensated Liver Cirrhosis	Stem cell	Effect of Mesenchymal Stem Cells-derived Exosomes in Decompensated Liver Cirrhosis	NCT05871463
	Dystrophic Epidermolysis Bullosa	Stem cell	MSC EVs in Dystrophic Epidermolysis Bullosa	NCT04173650
	Dry Eye Disease	Stem cell	Safety and Efficacy of Pluripotent Stem Cell-derived Mesenchymal Stem Cell Exosome (PSC-MSC-Exo) Eye Drops Treatment for Dry Eye Diseases Post Refractive Surgery and Associated with Blepharospasm	NCT05738629
	Hair Loss, Alopecia	Stem cell	Exosome Effect on Prevention of Hairloss	NCT05658094
	Premature Ovarian Insufficiency, Diminished Ovarian Reserve	Stem cell	A Proof of Concept Study to Evaluate Exosomes from Human Mesenchymal Stem Cells in Women with Premature Ovarian Insufficiency (POI)	NCT06072794
	Healthy	ILB-202 exosomes	A Study to Evaluate the Safety and Tolerability of ILB-202	NCT05843799
	Refractory Depression and Anxiety Disorder, Neurodegenerative Diseases	Not-specified	Focused Ultrasound and Exosomes to Treat Depression, Anxiety, and Dementias	NCT04202770
	Myocardial Infarction and Ischemia	Stem cell	Co-transplantation of Mesenchymal Stem Cell Derived Exosomes and Autologous Mitochondria for Patients Candidate for CABG Surgery	NCT05669144

	Heart Failure	Stem cell	Treatment of Non-ischemic Cardiomyopathies by Intravenous Extracellular Vesicles of Cardiovascular Progenitor Cells	NCT05774509
	Metabolic Syndrome, Healthy Volunteers	Plant	The Role of a Natural Product, Containing Nanovesicles From Citrus Limon (L.) Juice, on Different CV Risk Factors	NCT04698447
	Percutaneous Coronary Intervention	Blood	Safety Evaluation of Intracoronary Infusion of Extracellular Vesicles in Patients Following Coronary Stent Implantation	NCT04327635
	Bronchopulmonary Dysplasia	Stem cell	A Safety Study of IV Stem Cell-derived Extracellular Vesicles (UNEX-42) in Preterm Neonates at High Risk for BPD	NCT03857841
	Infertility	Platelet	Use of Autologous Exosomes vs Platelet Growth Factors to Regenerate the Ovary in Women with Infertility	NCT06773572
	Autism Spectrum Disorder	Stem cell	Combined Photo-Biomodulation At Acupuncture Points, Autologous PRP, and Umbilical Cord-Derived Exosome Therapy in Autism Spectrum Disorder	NCT06600529
	Thinning Hair	Stem cell (Benev)	Study Evaluating the Efficacy and Safety of BENEV Exosome Regenerative Complex+ for Self-perceived Thinning Hair	NCT06571799
	Fistula Perianal	Stem cell	Human Placenta Mesenchymal Stem Cells Derived Exosomes Injection for Treatment of Complex Anal Fistula	NCT06568653
	Dry Eye Syndromes	Stem cell	Limbal Stem Cell Derived Exosome (LSC-Exo) Eye Drop for Treatment of Dry Eye	NCT06543667
	Pilonidal Sinus, Pilonidal Disease	Stem cell	The Role of Mesenchymal Stem Cell and Exosome in Treating Pilonidal Sinus Disease in Children	NCT06391307
	Safety evaluation	Platelet (PEP; Purified Exosome Product)	Purified Exosome Product (PEP) Injected into the Hypodermis	NCT06429033
	Bronchopulmonary Dysplasia	Stem cell	Safety and Efficacy of MSC-EVs in the Prevention of BPD in Extremely Preterm Infants	NCT06279741
	Sudden Deafness, Sensorineural Hearing Loss	Autologous blood monocyte	Autologous Blood Monocyte Vesicles for the Treatment of Sudden Deafness	NCT06707389
	Hearing Loss	Extracellular Vesicle-enriched Secretome Fraction (VSF1.01)	Intracochlear Application of VSF1.01 for the Reduction of Cochlear Implant Surgery Related Trauma	NCT06545175

**EVs used for diagnostic and treatment follow-up approaches (Interventional clinical trials)**			
**Disease group**	**Condition**	**Study Title**	**NCT Number**
Covid	Covid19	Treatment of SARS-CoV-2 Virus Disease (COVID-19) in Humans with Hemopurifier Device	NCT04595903
Endocrine and metabolic disorders	Diabetes Mellitus, Type 2	Efficacy of Educational Nutrition and Exercise on the Regulation of Appetite Through Exosomes in Type 2 Diabetics	NCT05259449
	Endothelial Dysfunction	Circulating Exosomes and Endothelial Dysfunction in Patients with Obstructive Sleep Apneas Hypopneas Syndrome	NCT04459182
	Insulin Resistance	Effect of Dietary Protein on the Regulation of Exosome microRNA Expression in Patients with Insulin Resistance.	NCT05318898
	Obstructive Sleep Apnea	Extracellular microRNA: Biomarkers of Endothelial Dysfunction in Obese Adolescents & Adults with Obstructive Sleep Apnea	NCT03546751
	Diabetes Mellitus, Type 2	Identification and Validation of Noninvasive Biomarkers of the Diagnosis and Severity of NASH in Type 2 Diabetics	NCT03634098
	Obesity, Insulin Resistance	Small Extracellular Vesicles and Insulin Action	NCT05933707
	BMI <25 or >30	Training Induced Muscle Exosome Release	NCT04500769
	Obesity, Weight Loss	Adipocyte-Derived Extracellular Vesicles, Weight Loss, and Endothelial Function	NCT06776081
	Diabetes, Exercise, Lifestyle Intervention	Differential Regulation of miRNA and Protein in Human Plasma Extracellular Vesicles by Different Types of Exercise	NCT06609785

	Type 2 Diabetes, Obesity	Extracellular Vesicles, Insulin Action, and Exercise	NCT06546085
	Obesity	Circulating Microvesicles Regulating Metabolic Homeostasis in Obesity After Caloric Restriction Programs	NCT06395246
	Obesity, Metabolic Syndrome	Deciphering the Role of Dietary Fatty Acids on Extracellular Vesicles-mediated Intercellular Communication	NCT06051461
Inflammatory and autoimmune conditions	HIV Infection	Inflammation, NK Cells, Antisense Protein and Exosomes, and Correlation with Immune Response During HIV Infection	NCT05243381
	Allergic Asthma, Severe Eosinophilic Asthma	Non-coding RNAs Analysis of Eosinophil Subtypes in Asthma	NCT04542902
Malignancies	Non-Small Cell Lung Cancer	Prediction of Immunotherapeutic Effect of Advanced Non-small Cell Lung Cancer	NCT04427475
	Glioma	Clinical Relevance of Detecting Molecular Abnormalities in Glial Tumor Exosomes	NCT06116903
	Prostatic Neoplasms	18F-DCFPyL Positron Emission Tomography (PET)/Computed Tomography (CT) in Men with Prostate Cancer	NCT03824275
	Non-Small Cell Lung Cancer	Exosomes Detection for the Prediction of the Efficacy and Adverse Reactions of Anlotinib in Patients with Advanced NSCLC	NCT05218759
	Breast Cancer, Leptomeningeal Metastasis	Analyses of Exosomes in the Cerebrospinal Fluid for Breast Cancer Patients with Suspicion of Leptomeningeal Metastasis.	NCT03974204
	Non-Small Cell Lung Cancer	Clinical Research for the Consistency Analysis of PD-L1 in Lung Cancer Tissue and Plasma Exosome Before and After Radiotherapy	NCT02869685
	Non-Small Cell Lung Cancer	Clinical Research for the Consistency Analysis of PD-L1 in Cancer Tissue and Plasma Exosome	NCT02890849
	Pancreatic Carcinoma	Ultra-High Resolution Optical Coherence Tomography in Detecting Micrometer Sized Early Stage Pancreatic Cancer in Participants With Pancreatic Cancer	NCT03711890
	Squamous Cell Carcinoma of the Head and Neck	Hemopurifier Plus Pembrolizumab in Head and Neck Cancer	NCT04453046
	Head and Neck Cancer	Metformin Hydrochloride in Affecting Cytokines and Exosomes in Patients with Head and Neck Cancer	NCT03109873
	Prostate Cancer	Evaluation of Fapi-pet in Prostate Cancer.	NCT05192694
	Non-Small Cell Lung Cancer	Olmutinib Trial in T790M (+) NSCLC Patients Detected by Liquid Biopsy Using BALF Extracellular Vesicular DNA	NCT03228277
	Metastatic Melanoma	Study of Molecular Mechanisms Implicated in the Pathogenesis of Melanoma. Role of Exosomes	NCT02310451
	Pancreas Adenocarcinoma	Circulating Extracellular Exosomal Small RNA as Potential Biomarker for Human Pancreatic Cancer	NCT04636788
	Triple Negative Breast Cancer, HER2-positive Breast Cancer	Exosome as the Prognostic and Predictive Biomarker in EBC Patients	NCT05955521
	Breast Cancer	Feasibility of Exosome Analysis in Cerebrospinal Fluid During the Diagnostic Workup of Metastatic Meningitis (Exo-LCR)	NCT05286684
	Lung Cancer	Molecular Profiling of Exosomes in Tumor-draining Vein of Early-staged Lung Cancer	NCT04939324
	Cancer	Impact of Group Psychological Interventions on Extracellular Vesicles in People Who Had Cancer	NCT04298398
	Advanced Breast Cancer	Omic Technologies to Track Resistance to Palbociclib in Metastatic Breast Cancer	NCT04653740
	Metastatic Breast Cancer	Genetic Characteristics of Metastatic Breast Cancer Patients	NCT04258735
	Small Cell Lung Cancer	Circulating EV Long RNA Profiles in SCLC	NCT05191849
	Cancer	The Modulatory Role of Internet MBCT on Extracellular Vesicles and Distress in Cancer Patients - Study Protocol	NCT04727593
	Meningioma	Plasma Extracellular Vesicles in Meningioma Patients	NCT06104930

	Prostatic Neoplasm	Clinical, Genetic and Environmental Determinants of Prostate Cancer Progression.	NCT06046131
	Cancer	Pimo Study: Extracellular Vesicle-based Liquid Biopsy to Detect Hypoxia in Tumours	NCT03262311
	Lymphoma, Aggressive Non-Hodgkin (B-NHL)	Exosomes and Immunotherapy in Non-Hodgkin B-cell Lymphomas	NCT03985696
	Non-Small Cell Lung Cancer	Neoadjuvant Lazertinib Therapy in EGFR-Mutation Positive Lung Adenocarcinoma Detected by BALF Liquid Biopsy	NCT05469022
	Prostate Cancer	Plasma Exosome RNA to Diagnose Prostate Cancer	NCT06604130
Neurological disorders	Alzheimer Disease	Combined Aerobic Exercise and Cognitive Training in Seniors at Increased Risk for Alzheimer's Disease	NCT05163626
	Drug Resistant Epilepsy	Circulating microRNAs as Biomarkers of RESPIratory Dysfunction in Patients with Refractory epilePSY	NCT03419000
	Acute Ischemic Stroke	Randomized Controlled Trial of Time-Restricted Feeding (TRF) in Acute Ischemic Stroke Patients	NCT04184076
	Mild Cognitive Impairment	Curcumin and Yoga Therapy for Those at Risk for Alzheimer's Disease	NCT01811381
	Parkinson´s Disease	Effect of a Progressive Treadmill Training Protocol for Parkinson's Disease	NCT05902065
	Myalgic Encephalomyelitis, Chronic Fatigue Syndrome	Analysis of Post-exertional Malaise Using a Two-day CPET in People With ME/CFS	NCT04026425
	Alzheimer Disease, Mild Cognitive Impairment	Phenserine on the Alzheimer's Treatment Horizon, Study 1	NCT06774261
	Immune Effector Cell Associated Neurotoxicity Syndrome	Temporal Characterization of Extracellular Vesicles During Cellular Therapy Using CAR-T Cells and During the Occurrence of Immune Effector Cell-Associated Neurotoxicity Syndrome	NCT06706102
Other	Blood Coagulation, Platelet Function	Effect of Exosomes Derived from Red Blood Cell Units on Platelet Function and Blood Coagulation	NCT02594345
	Healthy	Exosome Proteomics to Detect EPO	NCT03700515
	Sports Drug Abuse	The Effect of Micro-doses Erytropoietin on Exercise Capacity in Male and Females	NCT04965961
	Prehypertension	When the Kidney Reacts to Nutritional Changes	NCT04142138
	Postoperative Delirium, Circadian Rhythm Disorders	The Disorder of Circadian Clock Gene and Early Cognitive Dysfunction After General Anesthesia	NCT04421872
	Atrial Fibrillation	Role of Exosomes Derived from Epicardial Fat in Atrial Fibrillation	NCT03478410
	Exercise	Exercise-induced Changes in Exosomes	NCT05616234
	Healthy	The Influence of High and Low Salt on Exosomes in the Urine	NCT02823613
	Normal Cellular Metabolism	In Vivo Assessment of Cellular Metabolism in Humans	NCT02748369
	Smoking	Smoker Extracellular Vesicles Influence on Human Bronchial Epithelial Cells	NCT03608293
	Myocardial Infarction	Antiplatelet Therapy Effect on Extracellular Vesicles in Acute Myocardial Infarction	NCT02931045
	Anxiety Disorders	Trial with the Treatment of Sertraline in Youth with Generalized, Separation and/or Social Anxiety Disorders.	NCT04221997
	Muscle Atrophy	Mechanisms Underlying Local and Systemic Effects of Massage	NCT04131712
	Chronic Renal Failure	Extracellular Vesicles as Biomarkers for Chronic Renal Failure	NCT04700631
	Arterial stiffness	Extracellular Vesicles and Endothelial Function in Transgender Subjects	NCT04971447

	Cardiovascular health/disease	Fish Oil-derived N-3 Polyunsaturated Fatty Acids and Extracellular Vesicles	NCT03203512
	Exercise Training	Impact of Exercise Intensity on Fitness, Extracellular Vesicles, Inflammation, and Metabolism	NCT06398548
	Sarcopenia, Anabolic Resistance	Strength Training and Resveratrol	NCT06585865
	Healthy Volunteers, Energy Metabolism	Exercise-regulated Organ Crosstalk, Influence of IL-6	NCT06334653

**Observational clinical trials**			
**Disease group**	**Condition**	**Study Title**	**NCT Number**
Covid	Covid19	Immune Modulation by Exosomes in COVID-19	NCT05191381
Endocrine and metabolic disorders	Diabetic Nephropathy	Expression Analysis of Urinary Exosome in Type 2 Diabetic Kidney Disease and Evaluation of Its Clinical Diagnostic Value	NCT06123871
	Diabetic Retinopathy	Study on Exosome Changes in Patients with Proliferative Diabetic Retinopathy	NCT06198543
	Diabetic Nephropathy	Expression Analysis of Urinary Exosome in Type 2 Diabetic Nephropathy and Evaluation of Its Clinical Diagnostic Value	NCT06097351
	Diabetic Retinopathy	Proteomic Study of Plasma Exosomes in Patients with Diabetic Retinopathy	NCT06188013
	Oocyte Maturation	Follicular Fluid Exosome miRNA During Oocyte Maturation	NCT04382872
	Pancreatogenic Type 3C Diabetes Mellitus	Evaluation of OGTT in Patients with Post-pancreatitis Diabetes Mellitus for Diagnosis and Characterization	NCT05989867
	Diabetic Retinopathy	Role of the Serum Exosomal miRNA in Diabetic Retinopathy (DR)	NCT03264976
	Gaucher Disease	Extracellular Vesicles as Potential Biomarkers and Therapeutic Target in Gaucher Disease	NCT05843552
	Type 1 and 2 Diabetes Mellitus	Circulating Extracellular Vesicles Released by Human Islets of Langerhans	NCT03106246
	Newborn Metabolic Bone Diseases	Exosome microRNAs as Potential Biomarkers of Metabolic Bone Disease of Prematurity	NCT06368154
	Obesity	Differences in Extracellular Vesicles from Adipose Tissue of Individuals with Obesity	NCT06444646
	Type 2 Diabetes Mellitus-Associated Erectile Dysfunction Following Radical Prostatectomy	Regenerative Injection of Stem Cells or Stem Cell-derived Exosomes for Erectile Dysfunction (RISE)	NCT06605508
	Glucose Intolerance, Insulin Resistance, Diabetes	Extracellular Vesicle Cargo in Obesity and Type 2 Diabetes	NCT06401876
Inflammatory and autoimmune conditions	Sepsis	Dendritic Cells-Derived Exosomes in Human Sepsis	NCT02957279
	Thyroid Diseases	Early Detection of Autoimmune Thyroid Heart Disease Via Urinary Exosomal Proteins	NCT03984006
	Sepsis	Function of Circulating Exosomes in Sepsis-induced Immunosuppression	NCT04979767
	Lupus Nephritis	Serological and Urinary Biomarkers in Latin American Patients with Systemic Lupus Erythematosus: GLADEL 2.0 Cohort	NCT04534647
	Lupus Nephritis	Urine Exosomes to Identify Biomarkers for LN	NCT04894695
	Kidney Transplantation	Major Activation of NCC in Graft Urinary Exosomes	NCT03503461
	Kidney Transplant Failure and Rejection	Urinary Transglutaminase 2 as a Biomarker for Kidney Allograft Fibrosis	NCT03487861

	Hemodynamic Instability	A Study of Exosome Proteomics and Hemodynamics in Sepsis	NCT03267160
	Sepsis	Changes of Exosomes and Biomarkers in Plasma and Alveolar Lavage Fluid of Patients with Sepsis Complicated With ARDS	NCT05476029
	Myasthenia Gravis	Screening of Serum Exosomal miRNA as a Biomarker for Ocular Muscle Myasthenia Gravis	NCT05888558
	Heart Transplant Rejection	Research on Patients with Heart Transplantation	NCT04921774
	Otitis Media	The Effects of Exosomes in Otitis Media with Effusion	NCT05402267
	Sepsis	Study of MSC-Exo on the Therapy for Intensively Ill Children	NCT04850469
	Osteoarthritis	Effects of ASC Secretome on Human Osteochondral Explants	NCT04223622
	Late-Onset Neonatal Sepsis	Markers of Platelet Activation foR Identification of Late Onset Sepsis in Preterm Infants	NCT05530330
	HIV	Sequential Analysis Before and After Treatment Initiation to Unravel the Role of Naturally Occurring Extracellular Vesicles in HIV Infection	NCT04653610
	Heart Transplant Rejection	Biomarkers for Diagnosis, Prognosis, and Targeted Therapy After Heart Transplantation	NCT06064123
	Dry Eye Syndrome (DES), Sjogren's Syndrome, Xerophthalmia	Exosomes Proteomic for Sjogren's Syndrome and Dry Eye Syndrome	NCT06771427
	Neonatal Jaundice from Other Specified Causes, ABO Incompatibility, ABO Hemolytic Disease	The Role of Breastmilk and Serum Exosomes in Neonatal Jaundice Due to ABO Incompatibility	NCT06502847
	Dry Eye Syndrome (DES), Sjogren's Syndrome, Xerophthalmia	Exosome-miRNA and Transcriptome Profiling of Dry Eye Syndrome and Sjogren's Syndrome	NCT06475027
	Systemic Lupus Erythematosus Nephritis	Monitoring Lupus Nephritis Through Urinary Extracellular Vesicles	NCT06642402
	Acute Respiratory Distress Syndrome	Explore Potential Plasma and BALF Immunometabolic and Lipidomic Biomarkers for Identifying ARDS Endotypes	NCT05451342
	Acute Respiratory Distress Syndrome	Association Between the Level of EV-TF and the Occurence of Pulmonary Embolism in Patients With ARDS	NCT05855317
	Acute Respiratory Distress Syndrome	The Mechanism of Extracellular Vesicles Containing Mitochondrial DNA in ARDS Lung Injury Caused by Extrapulmonary Sepsis	NCT05061212
Malignancies	Gastrointestinal Cancer	Prospectively Predict the Efficacy and Explore the Mechanism of Treatment of Gastrointestinal Tumors Based on Peripheral Multi-omics Liquid Biopsy	NCT05427227
	Pancreatic Cancer	Acquisition of Portal Venous CTCs and Exosomes from Patients with Pancreatic Cancer by EUS	NCT03821909
	Lung Cancer	Improving the Early Detection of Lung Cancer by Combining Exosomal Analysis of Hypoxia with Standard of Care Imaging	NCT04629079
	Breast Neoplasms	A Pilot Study of Tumor-Derived Exosomes as Diagnostic and Prognostic Markers in Breast Cancer Patients Receiving Neoadjuvant Chemotherapy	NCT01344109
	Pulmonary Nodules	Clinical Study of ctDNA and Exosome Combined Detection to Identify Benign and Malignant Pulmonary Nodules	NCT04182893
	Prostate Cancer	Clinical Validation of a Urinary Exosome Gene Signature in Men Presenting for Suspicion of Prostate Cancer	NCT02702856
	Prostate Cancer, Urothelial Carcinoma	Glycosylation of Exosomes in Prostate and Urothelial Carcinoma	NCT04960956
	Rectal Cancer	Exosomes in Rectal Cancer	NCT03874559
	Hepatocellular Carcinoma	Clinical Study for Combined Analysis of CTC and Exosomes on Predicting the Efficacy of Immunotherapy in Patients with Hepatocellular Carcinoma	NCT05575622
	Prostate Cancer	How Does Prostate Cancer Metastasize? Studying the Role of Secreted Packages (Exosomes) From Fat Tissue in Lean and Obese Patients	NCT04167722
	Gastric Cancer	Circulating Exosomes as Potential Prognostic and Predictive Biomarkers In Advanced Gastric Cancer Patients ("EXO-PPP Study")	NCT01779583

	Sarcoma	Study of Exosomes in Monitoring Patients with Sarcoma (EXOSARC)	NCT03800121
	Pancreatic cancer	Tumor Exosome Liquid Biopsy Strategy to Diagnose Pancreatic Cancer	NCT06108531
	Prostate Cancer	ExoDx Prostate Evaluation in Prior Negative Prostate Biopsy Setting	NCT04357717
	Lung Cancer	Multicenter Clinical Research for Early Diagnosis of Lung Cancer Using Blood Plasma Derived Exosome	NCT04529915
	Cholangiocarcinoma	ncRNAs in Exosomes of Cholangiocarcinoma	NCT03102268
	Bone Metastases	Identification and Characterization of Predictive Factors of Onset of Bone Metastases in Cancer Patients	NCT03895216
	Breast Cancer	A Study to Measure the Expression of the HER2-HER3 Dimer in Tumour and Blood (Exosomes) Samples from Patients with HER2 Positive Breast Cancer Receiving HER2 Targeted Therapies	NCT04288141
	Gallbladder Carcinoma	A Study of Circulating Exosome Proteomics in Gallbladder Carcinoma Patients	NCT03581435
	Thyroid Cancer	Correlation Between Various Urinary Exosomal Protein Biomarkers and Pathological Manifestation in Thyroid Follicular Neoplasm: Early and Pre-operative Diagnosis of Follicular Thyroid Cancer	NCT05463107
	Colorectal Cancer	Identification of New Diagnostic Protein Markers for Colorectal Cancer	NCT04394572
	Rectal Cancer	A Prospective Feasibility Study Evaluating Extracellular Vesicles Obtained by Liquid Biopsy for Neoadjuvant Treatment Response Assessment in Rectal Cancer	NCT04852653
	Lung Cancer	Circulating and Imaging Biomarkers to Improve Lung Cancer Management and Early Detection	NCT04315753
	Lung Cancer	Combined Diagnosis of CT and Exosome in Early Lung Cancer	NCT03542253
	Colorectal Cancer	Contents of Circulating Extracellular Vesicles: Biomarkers in Colorectal Cancer Patients	NCT04523389
	New Tumor Diagnostics from Human Plasma Samples	Cell Free Circulating Nucleic Acids as New Tumor Diagnostics from Human Plasma Samples	NCT04081194
	Non-Small Cell Lung Cancer	The Study of Exosome EML4-ALK Fusion in NSCLC Clinical Diagnosis and Dynamic Monitoring	NCT04499794
	Lymph Node Metastasis	A Prospective, Multicenter Cohort Study of Urinary Exosome lncRNAs for Preoperative Diagnosis of Lymphatic Metastasis in Patients with Bladder Cancer	NCT05270174
	Thyroid Cancer	Urinary Exosomal Biomarkers of Thyroglobulin and Galectin-3 for Prognosis and Follow-up in Patients of Thyroid Cancer	NCT04948437
	Prostate Cancer	Prostasomes as Diagnostic Tool for Prostate Cancer Detection	NCT03694483
	Pancreatic Cancer	Interrogation of Exosome-mediated Intercellular Signaling in Patients with Pancreatic Cancer	NCT02393703
	Lung Cancer	Comparision of Various Biomarkers Between Peripheral and Pulmonary Blood	NCT05587114
	Oropharyngeal Cancer	Exosome Testing as a Screening Modality for Human Papillomavirus-Positive Oropharyngeal Squamous Cell Carcinoma	NCT02147418
	Gastric Cancer	Use of a Liquid Biopsy Signature to Detect Early-onset Gastric Cancer	NCT06023121
	Pancreas Cancer	ExoLuminate Study for Early Detection of Pancreatic Cancer	NCT05625529
	Thyroid Cancer	Predicting Prognosis and Recurrence of Thyroid Cancer Via New Biomarkers, Urinary Exosomal Thyroglobulin and Galectin-3	NCT03488134
	Ovarian Cancer	Non-coding RNA in the Exosome of the Epithelia Ovarian Cancer	NCT03738319
	Lung Cancer	Serum Exosomal Long Noncoding RNAs as Potential Biomarkers for Lung Cancer Diagnosis	NCT03830619
	Osteosarcoma, Pulmonary Metastases	Construction of Microfluidic Exosome Chip for Diagnosis of Lung Metastasis of Osteosarcoma	NCT05101655

	Clear Cell Renal Cell Carcinoma	Evaluation of Urinary Exosomes Presence from Clear Cell Renal Cell Carcinoma	NCT04053855
	Osteosarcoma, Lung Metastases	Circulating Exosome RNA in Lung Metastases of Primary High-Grade Osteosarcoma	NCT03108677
	Bladder Cancer, Urothelial Carcinoma	Research on the Accurate Diagnosis of Urinary Tract Tumors and the Development of Kits	NCT06193941
	Renal Cell Carcinoma	A Companion Diagnostic Study to Develop Circulating Exosomes as Predictive Biomarkers for the Response to Immunotherapy in Renal Cell Carcinoma	NCT05705583
	Rectal Neoplasm Malignant Carcinoma	Study on Predictive Biomarkers of Neoadjuvant Chemoradiotherapy for Rectal Cancer	NCT04227886
	Melanoma	Analysis of Circulating Exosomes in Melanoma Patients	NCT05744076
	Lung Cancer with Central Nervous System Metastasis	Non-small Cell Lung Cancer with Central Nervous System Metastasis	NCT06026735
	Urologic Cancer	Clinical Evaluation of ExoDx Prostate(IntelliScore) in Men Presenting for Initial Prostate Biopsy	NCT04720599
	Pancreatic Ductal Adenocarcinoma (PDAC)	Diagnostic Accuracy of Circulating Tumor Cells (CTCs) and Onco-exosome Quantification in the Diagnosis of Pancreatic Cancer - PANC-CTC	NCT03032913
	Thyroid Cancer	Anaplastic Thyroid Cancer and Follicular Thyroid Cancer-derived Exosomal Analysis Via Treatment of Lovastatin and Vildagliptin and Pilot Prognostic Study Via Urine Exosomal Biological Markers in Thyroid Cancer Patients	NCT02862470
	Thyroid Gland Carcinoma	Development of Liquid Biopsy Technologies for Noninvasive Cancer Diagnostics in Patients with Suspicious Thyroid Nodules or Thyroid Cancer	NCT04742608
	Breast Cancer	Extracellular Vesicles in Breast Cancer Patientsin Undergone Neoadjuvant Chemotherapy	NCT05831397
	Oral Premalignant Lesions	The Senstivity and Specificity of Using Salivary miRNAs in Detection of Malignant Transformation of Oral Lesions.	NCT04913545
	Non-Small Cell Lung Cancer	Extracellular Vesicles and Particles (EVP) as Biomarkers of Recurrence in Non-Small Cell Lung Cancer	NCT05424029
	Breast Cancer	Clinical Study of Glycosylated Extracellular Vesicles for Early Diagnosis of Breast Cancer	NCT05417048
	Breast Cancer	Characterization of Extracellular Vesicles in Breast Cancer Patients	NCT05798338
	Advanced Gastric Adenocarcinoma	Prospectively Predict the Efficacy of Treatment of Gastrointestinal Tumors Based on Peripheral Multi-omics Liquid Biopsy	NCT04993378
	Retinoblastoma	New Strategies to Detect Cancers in Carriers of Mutations in RB1	NCT04164134
	Pancreatic Cancer	Role of Exosomes in Pancreatic Cancer Progression	NCT06777030
	Lung Adenocarcinoma, EGFR Activating Mutation	Plasma Exosomes Reveal the Efficacy of Targeted Therapy in Patients with EGFR Mutation in Lung Adenocarcinoma	NCT06730477
	Gastric Cardia Cancer	Integrative Analysis of Exosome-Mediated Single-Cell Transcriptomics and Proteomics in Gastric Cardia Cancer	NCT06702891
	Colorectal Cancer	Exosome-based Detection of Molecular Residual Disease in Stage II-III Colorectal Cancer	NCT06654622
	Oral Leukoplakia, Oral Cancer	Saliva and Plasma Exosomes for Oral Leukoplakia Malignant Transformation Diagnosis and Oral Cancer Prognosis Monitoring	NCT06469892
	Pancreatic Ductal Adenocarcinoma, Pancreatic Cancer	Pancreatic Cancer Detection Consortium	NCT06388967
	Cholangiocarcinoma	Detecting Lymph Node Metastasis in Intrahepatic Cholangiocarcinoma (LyMIC)	NCT06381648
	Gastric Cancer, Gastric Adenocarcinoma	Stomach Cancer Exosome-based Detection	NCT06342427
	Colorectal Cancer, Colorectal Adenocarcinoma	Early Onset Colorectal Cancer Detection	NCT06342401
	Colorectal Cancer/Poly/Dysplasia/Adenoma	Early Detection of Advanced Adenomas and Colorectal Cancer	NCT06342440

	Hepatocellular Carcinoma, Intrahepatic Cholangiocarcinoma	An Exosome-Based Liquid Biopsy for the Differential Diagnosis of Primary Liver Cancer	NCT06342414
	Gastric Cancer, Esophagus Cancer	Exosome-based Liquid Biopsies for Upper Gastrointestinal Cancers Diagnosis	NCT06278064
	Hairy Cell Leukemia (HCL)	Decoding the Extracellular Vesicles-driven Communication in the Microenvironment of Hairy Cell Leukemia to Improve Patient Care Management	NCT06774677
	CAR-T Cell Therapy	Study of Extracellular Vesicles (EV) in Patients Undergoing CAR-T Cell Therapies	NCT06554951
	Breast Neoplasm	A Prospective Study to Develop and Clinically Validate an in Vitro Diagnostic Medical Device That Uses Blood to Classify Patients at High Risk for Breast Cancer	NCT06672302
	Ovarian Cancer	Exosome-based OCS Scores for Predicting Ovarian Cancer Recurrence	NCT06558019
	Hairy Cell Leukemia (HCL)	Hairy Cell Leukemia: Harnessing the Full Power of Extracellular Vesicles to Improve Patient Care Management	NCT06764524
	Prostate Carcinoma	Determination of Baseline Levels for Prostate Cancer-Derived Extracellular Vesicles Following Local Treatment of Prostate Cancer	NCT06326216
	Cancer	Spectroscopic Profiling of Extracellular Vesicles by Resonant Gold Nanostructures in the Infrared	NCT06266195
Neurological disorders	Neurocognitive Disorder, Mild Cognitive Impairment	The University of Hong Kong Neurocognitive Disorder Cohort	NCT03275363
	Neurodevelopmental Disabilities	Emotional Regulation in Children With ND: The Role of Genomic Variation, Proteomic Patterns, and Early Experience	NCT05004090
	Stroke	Extracellular Vesicles as Stroke Biomarkers	NCT05370105
	Intracerebral Hemorrhage	Application of Circulating Exosomes in Early Diagnosis and Prognosis Evaluation After Intracerebral Hemorrhage	NCT05035134
	Parkinson's Disease	LRRK2 and Other Novel Exosome Proteins in Parkinson's Disease	NCT01860118
	Parkinson's Disease	Fox BioNet Project: ECV-003	NCT03775447
	Parkinson's Disease	FoxBioNet: ECV (Extracellular Vesicle) 004	NCT04603326
	Alzheimer Disease	Ectosomes, New Biomarkers of Tau Pathology?	NCT03381482
	Parkinson's Disease and Parkinsonism	Saliva and Extracellular Vesicles for Parkinson's Disease	NCT05320250
	Ischemic Stroke, Transient Ischemic Attack	Extracellular Vesicles and Dysregulated Coagulation in the Prediction of Stroke	NCT05645081
	Huntington Disease	Extracellular Vesicles for HD	NCT06082713
	Traumatic Brain Injury	Application of Circulating Extracellular Vesicles in Early Disease Assessment and Prognosis After Traumatic Brain Injury	NCT05279599
	Stroke, TIA, Stroke-mimics	Extracellular Vesicle Surface Markers in Acute Cerebrovascular Syndromes	NCT06319742
	Ischemic Stroke, Transient Ischemic Attack	Vascular Cognitive Decline and Dementia	NCT06257823
Other	Myocardial Infarction	Differential Expression and Analysis of Peripheral Plasma Exosome miRNA in Patients with Myocardial Infarction	NCT04127591
	Sleep Apnea Syndromes	Exosomes Implication in PD1-PD-L1 Activation in OSAS	NCT03811600
	Delirium	Exploring of Serum Biomarkers of Delirium After Cardiovascular Surgery	NCT06007755
	Chronic Kidney Disease of Unknown Etiology (CKDu)	An Investigation into the Cardiovascular Risk and Aetiology of CKDu in Sri Lanka	NCT02226055
	Exercise Physiology	Performance Determinants Factors in Elite Endurance Athletes.	NCT03569566

	Bipolar Affective and Major Depressive Disorder	Exosome for Early Diagnosis of Bipolar Affective Disorder	NCT05915312
	Coronary Artery Bypass Surgery	Study of Role of Blood Microvescicles and Exosomes in Patients with Graft Occlusion After Aortocoronary Bypass Surgery	NCT05411445
	Acute Lung Injury	Omics Sequencing of Exosomes in Body Fluids of Patients with Acute Lung Injury	NCT05058768
	Preeclampsia	microRNAs Role in Pre-eclampsia Diagnosis	NCT03562715
	Hypertension	New Biomarkers and Difficult-to-treat Hypertension	NCT03034265
	Preeclampsia	Exosome Cargo from Preeclampsia Patients	NCT04154332
	Neonatal Opioid Withdrawal Syndrome	MicroRNA Biomarkers for Neonatal Opioid Withdrawal Syndrome	NCT05937594
	STEMI	ExosoMe as Integrative Tool for pRognostic Stratification of Adverse Cardiac remodeLing in stEmi Patients: the MIRACLE Study	NCT06070974
	Effect of Exercise	Effect of Acute Exercise on Exosome Associated Biomarkers	NCT05541133
	Analysis of Endometrium-derived EVs	Isolation and Characterization of the Extracellular Vesicles Secreted by the Human Endometrium	NCT02797834
	Coronary Artery Disease	Echocardiography: Value and Accuracy at REst and STress	NCT03674255
	Cardiovascular Diseases	Kinetics of cEVs Over the 24-hour Dosing Interval After Low-dose Aspirin Administration	NCT05584943
	End-stage Kidney Disease	Kinetics of Extracellular Vesicles in Hemodialysis	NCT05957146
	Pulmonary Hypertension	Platelet Reactivity and Treatment with Prostacyclin Analogues in Pulmonary Arterial Hypertension	NCT04578223
	Heart failure	Salivary Extracellular Vesicle Associated lncRNAs in Heart Failure (SEAL-HF)	NCT06169540
	Intensive Meditation in Novice and Experienced Meditators	Integrating Magnetic Imaging with Rich Phenotypes	NCT06615531
	Platelet Thrombus	Characterization of Exosomes Platelets-released	NCT06298682
	Extremely Low Gestational Age Newborns, Cardiovascular Immaturity	Cardiovascular Immaturity in Extremely Low Gestational Age Newborns: A Fundamental Prospective Study	NCT06737965
	Joint Disease	Extracellular Vesicles in Fibrin Gel for Cartilage Repair	NCT06713902
	Endometriosis, Adenomyosis	Next-Generation Endometriosis Diagnostics Through Comprehensive Multi-Dimensional Analysis	NCT06572852
	Cardiovascular Diseases	EVOC - EVs in Obesity and Cardiometabolic Disease	NCT06408961
	Ureteropelvic Junction Obstruction, Chronic Kidney Diseases	The PRO-FUTURE Project	NCT06382233
	Anorexia Nervosa	Biomarkers in EV Associated with Marrow Adiposity in Anorexia	NCT06712485
	Silent Myocardial Ischemia, Acute Myocardial Infarction	Silent Myocardial Ischemia in Patients Undergoing Non-Oncological Abdominal Surgeries	NCT06536686
	Congenital Heart Disease, Single-ventricle, Thrombosis	Extracellular Vesicle Micro RNA Profiling in Congenital Heart Disease: Fetal-Maternal Regulation in Neonatal Thrombosis	NCT06434207
	Preeclampsia	EV Based Platform for Monitoring Therapeutics Response During Pregnancy (ARISE)	NCT06249178
